# Choice of Voxel-based Morphometry processing pipeline drives variability in the location of neuroanatomical brain markers

**DOI:** 10.1038/s42003-022-03880-1

**Published:** 2022-09-06

**Authors:** Xinqi Zhou, Renjing Wu, Yixu Zeng, Ziyu Qi, Stefania Ferraro, Lei Xu, Xiaoxiao Zheng, Jialin Li, Meina Fu, Shuxia Yao, Keith M. Kendrick, Benjamin Becker

**Affiliations:** 1 The Center of Psychosomatic Medicine, Sichuan Provincial Center for Mental Health, Sichuan Provincial People’s Hospital, MOE Key Laboratory for Neuroinformation, High-Field Magnetic Resonance Imaging Key Laboratory of Sichuan Province, University of Electronic Science and Technology of China, Chengdu, China; 2 Institute of Brain and Psychological Sciences, Sichuan Normal University, Chengdu, China; 3 Neuroradiology Department, Fondazione Instituto Neurologico Carlo Besta, Milan, Italy

**Keywords:** Neuroscience, Biomarkers

## Abstract

Fundamental and clinical neuroscience has benefited tremendously from the development of automated computational analyses. In excess of 600 human neuroimaging papers using Voxel-based Morphometry (VBM) are now published every year and a number of different automated processing pipelines are used, although it remains to be systematically assessed whether they come up with the same answers. Here we examined variability between four commonly used VBM pipelines in two large brain structural datasets. Spatial similarity and between-pipeline reproducibility of the processed gray matter brain maps were generally low between pipelines. Examination of sex-differences and age-related changes revealed considerable differences between the pipelines in terms of the specific regions identified. Machine learning-based multivariate analyses allowed accurate predictions of sex and age, however accuracy differed between pipelines. Our findings suggest that the choice of pipeline alone leads to considerable variability in brain structural markers which poses a serious challenge for reproducibility and interpretation.

## Abstract (web summary)

Four common processing pipelines tested on two Voxel-based Morphometry (VBM) datasets yield considerable variations in results, raising issues on the interpretability and robustness of VBM results.

## Introduction

Human fundamental and clinical neuroscience aims to determine the contribution of specific brain systems to mental processes and disorders, and neuroimaging approaches have been widely employed to this end. Due to its high spatial resolution and noninvasive nature, Magnetic Resonance Imaging (MRI)-based assessments of brain structure and function have become one of the most widely used neuroimaging techniques. However, the complexity and flexibility of workflows in MRI analyses, and differences between the handful of commonly used analysis software packages, may lead to high variability in neuroimaging results^1^. This variability challenges the interpretation of the results with respect to the precise mapping of mental processes and brain-based biomarkers for mental disorders. Compared to the processing of functional MRI (fMRI) data, brain morphometry analyses of T1-weighted structural images allow less processing variations and may have higher test-retest reliability^1–6^. However, the choice of analytic software may still have a considerable impact on the results obtained. The variability in terms of whether and which specific brain regions pass the statistical threshold, in turn, impacts greatly on the interpretation of findings with respect to structure-function mapping or brain-based biomarkers and can significantly impede the sensitivity of subsequent neuroimaging meta-analyses.

Neuroanatomical research has benefited tremendously from the development of automated computational approaches such as Voxel-based Morphometry (VBM), examining variations in regional gray matter volume, and the more recently developed surface-based approaches (e.g., examining cortical thickness). VBM represents one of the most commonly used brain structural analytic approaches to date (e.g., a simple literature search using the term “voxel-based morphometry” or “VBM” on PubMed revealed 6210 studies, https://pubmed.ncbi.nlm.nih.gov, from 1993 to November 19, 2020, see also publications for VBM and other approaches such as “cortical thickness” and “surface area” in PubMed depicted in Fig. S1). The standardized and highly automated VBM workflow includes segmentation of gray matter from other brain tissues, normalization into standard stereotactic space, and smoothing with a Gaussian kernel before inferential statistics are applied. The corresponding inferential voxel-wise statistical models commonly determine (1) between-group differences in regional gray matter volume (GMV), e.g., between patients and controls or men and women^7–10^, or (2) associations between individual variations in regional GMV and behavioral phenotypes, including learning, age, or disorder-relevant traits^11–16^. Significant differences or associations are commonly interpreted in a regional-specific fashion, e.g., mapping specific behavioral functions to specific brain systems, and determining which brain regions undergo age-related changes or which regions contribute to mental disorders. More recently, machine-learning-based multivariate analytic approaches such as Multivariate Pattern Analyses (MVPA) have been increasingly applied to VBM data to detect subtle and spatially distributed patterns of brain structural variations to improve biomarker-based diagnostics of mental disorders^17–19^. MVPA aims at determining variations in the spatial pattern across multiple voxels simultaneously and is thus often more sensitive in detecting between-group differences or brain structural associations. The approach is based on training pattern recognition algorithms, for example, brain structural data, and can be applied to new data to predict group membership (e.g., patients vs. controls, or women vs. men) or individual variations in a continuous variable such as age.

A number of software packages have been developed and are widely utilized for VBM analyses. Among them, the currently most widely used ones are the Computational Anatomy Toolbox (CAT, www.neuro.uni-jena.de/cat), which is implemented in the Statistical Parametric Mapping software (SPM, https://www.fil.ion.ucl.ac.uk/spm/software/spm12/), and FSLVBM and FSLANAT, which are based on the FMRIB Software Library (FSL, https://fsl.fmrib.ox.ac.uk). To enhance the robustness and reproducibility of neuroimaging analyses, new modular preprocessing pipelines for structural MRI (e.g., sMRIPrep, https://www.nipreps.org/smriprep/) have been recently developed. Although the software packages generally employ similar processing steps to volumetric T1-weighted (anatomical) MRI data, differences in specific processing steps and their implementation exist. This raises the question of whether the choice of specific software and the application of software-specific default processing configurations may lead to variability in the results.

A recent study examined reliability and replicability in cortical thickness measures, using different software packages in large datasets of healthy subjects, and reported a similar cortical thickness distribution across software packages, although the absolute estimated values varied considerably among pipelines^20^. In contrast, studies exploring the replicability of VBM in samples of neurological patients revealed considerable variations among the processing pipelines, and results suggest that the VBM processing pipeline chosen strongly affects the clinical interpretation^21,22^. Specifically, spatial normalization inaccuracies and different spatial normalization templates and methods challenge one of the main assumptions of VBM, namely that individual brain differences and anatomical correspondence of brain areas are maintained during the spatial normalization process^23–27^. Moreover, VBM lacks a clear in vivo or ex vivo histological and neurobiological validation in humans^23^. Together this challenges the interpretation of VBM findings as biologically plausible markers for brain-based disorders or phenotypical variations.

Against this background, the present study systematically examined whether the choice of processing software influences the results of a VBM study. We included the most commonly used processing software packages (FSLVBM and FSLANAT as implemented in FSL v6.0, and CAT12.7, all recent releases) as well as an in-house pipeline using some sMRIPrep functionalities (version 0.6.2). The sMRIPrep pipeline served as an example of a customized pipeline based on different neuroimaging software packages. To model the typical scientific workflow, the recommended default configurations were employed to determine between-group differences and biological associations within two independent samples of healthy individuals (*n* = 200; *n* = 494). Given the previously reported low robustness of associations between psychological variables and brain structure see ref. ^28^, we focused on biological variables, i.e., sex and age^9,10,29–32^.

**Fig. 1:**
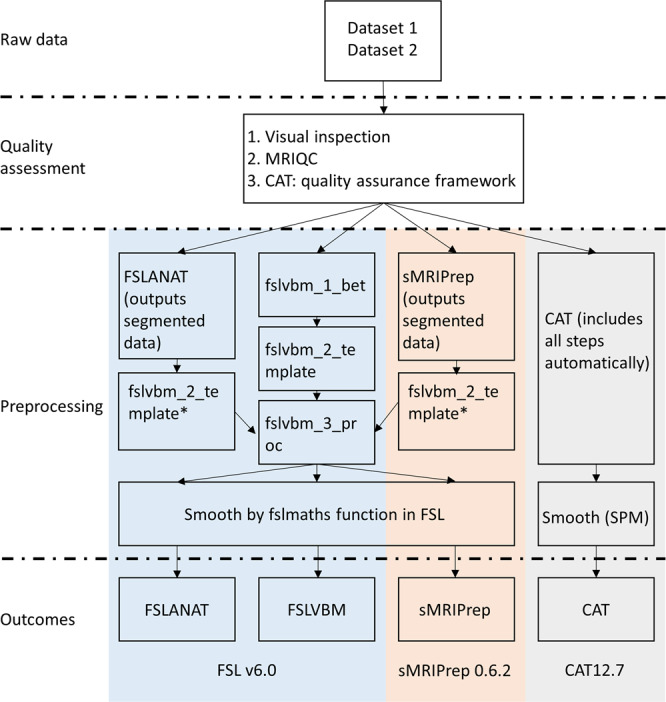
Overview flowchart of preprocessing steps across four pipelines. *Given that FSLANAT and sMRIPrep pipelines are mainly used for segmenting GM, WM, and CSF data, the segmented GM outcomes (in native space) were subjected to preprocessing steps from fslvbm_2_template (except for segmentation) and fslvbm_3_proc to produce normalized and modulated GM data.

To determine the effects of the choice of processing pipeline on the results of a typical VBM study, we examined sex differences and age-related changes with univariate analyses (group differences and regression, respectively) as well as multivariate analyses (machine-learning-based MVPA) in two large datasets (dataset 1, *n* = 200, age 18–26, 100 females; dataset 2, *n* = 494, age 19–80, 307 females) after processing the data with the commonly used VBM pipelines (Fig. 1). Specifically, the following systematic steps were conducted. First, spatial similarity and intraclass correlation (ICC, both voxel-wise and image-based estimations) were examined to determine the spatial similarity, homogeneity, and replicability of outcomes across pipelines before further statistical analyses. Second, results with respect to sex differences in GMV from univariate between-group comparisons between males and females were compared across pipelines. Third, results with respect to age-related GMV changes from univariate linear regression analysis were compared across pipelines. Finally, the effects of pipelines on multivariate prediction accuracy were examined by means of MVPA-based predictions of sex and age based on whole-brain GMV maps across pipelines.

## Results

### Spatial similarity and homogeneity within- and between-pipelines

We initially examined the spatial similarity maps of the preprocessed images within and between the pipelines (Fig. S2). Statistical analyses by means of ANOVA models with the repeated factor pipeline and the dependent variable spatial similarity in terms of z-transformed spatial correlation coefficients revealed a high variation in terms of pipeline and participants, both within- and between-pipelines (all tests Bonferroni’s corrected *p* < 0.01). For detailed ANOVA and post hoc results, please see supplemental material results, Fig. S3 and Tables S1–S9. Together these findings indicate significant spatial dissimilarities between GMV maps from the same participants between-pipelines as well as from the same pipeline between participants. Specifically, across the datasets, the lowest spatial similarity was observed between CAT and FSLVBM, and CAT and sMRIPrep, respectively. Notably, CAT reached a considerably higher within-pipeline spatial similarity as compared to the other pipelines (Fig. S3), reflecting a higher homogeneity of the processed GMV maps between participants when the data were processed with CAT.

### Cross-pipeline replicability

**Fig. 2:**
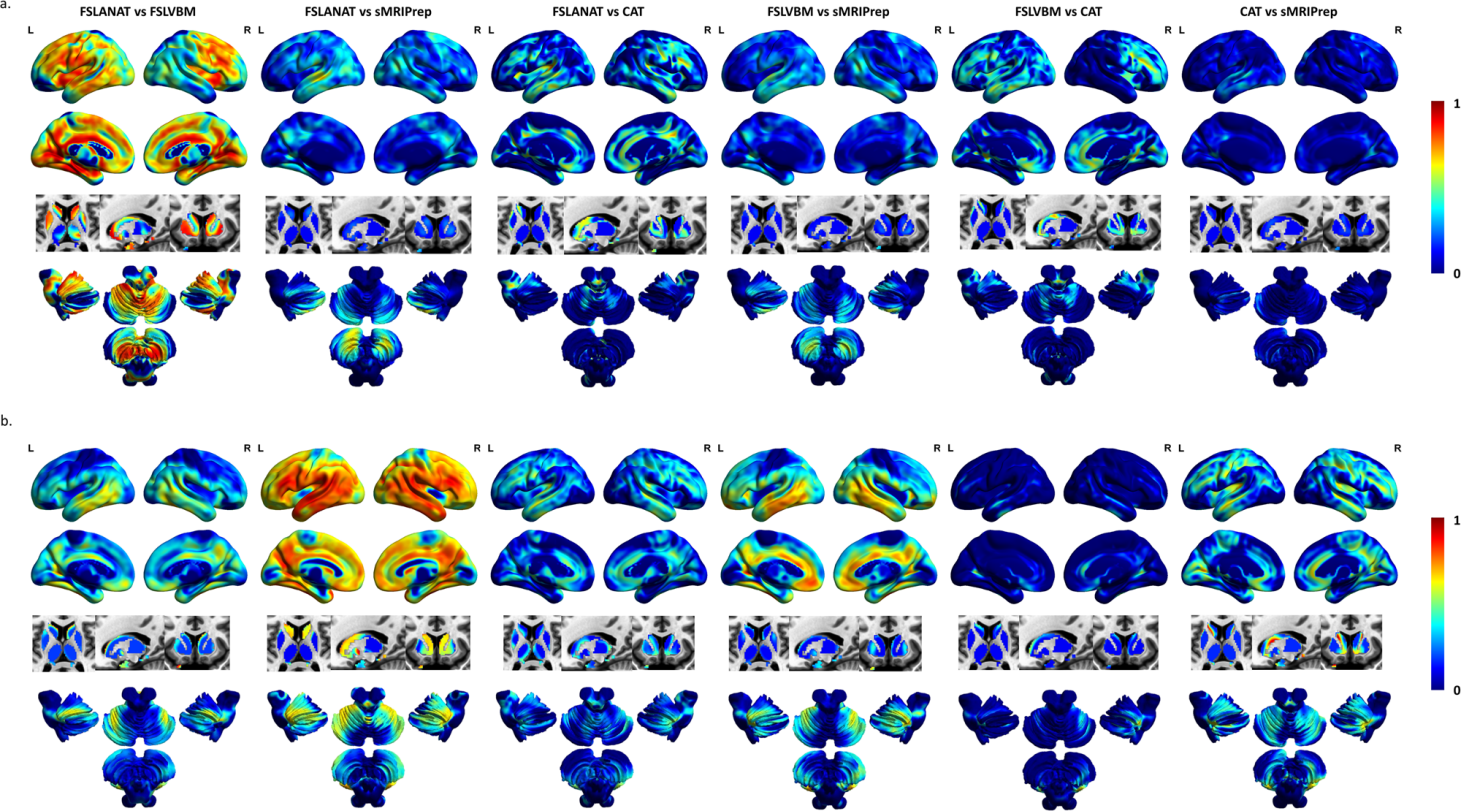
Voxel-level intraclass correlation coefficient (ICC) maps. Voxel-level intraclass correlation coefficient (ICC) maps between pipelines of **a** dataset 1 and **b** dataset 2. L left, R right. The color grading reflects the ICC value.

Examination of ICC maps for each between-pipeline comparison revealed generally low regional consistency between the GMV maps computed by different pipelines (see Fig. 2 for dataset 1 and 2). An exception was comparably high replicability between FSLANAT vs. FSLVBM in dataset 1, and FSLANAT vs. sMRIPrep in dataset 2 (Fig. 2). Of note, across the two datasets, the different pipelines exhibited relatively low between-pipeline replicability. This may be explained by the fact that the data were acquired in different imaging centers, MRI systems, and age range populations and suggests that complex interactions between the technological, sample, and analytic factors may contribute to variability see ref. ^33^. Examining the regional distribution of variations on the voxel level revealed that parietal and frontal regions in particular exhibited low consistency between pipelines. Examination of an image-based replicability index (I2C2) revealed a generally poor consistency between the pipelines (all image intraclass correlation coefficients < 0.4), confirming low inter-pipeline replicability. Only the replicability between CAT and FSLANAT or CAT and FSLVBM approached the ‘fair’ criterion (Table S10).

### Effects of the pipeline on univariate between-group statistical comparison: sex differences

**Fig. 3:**
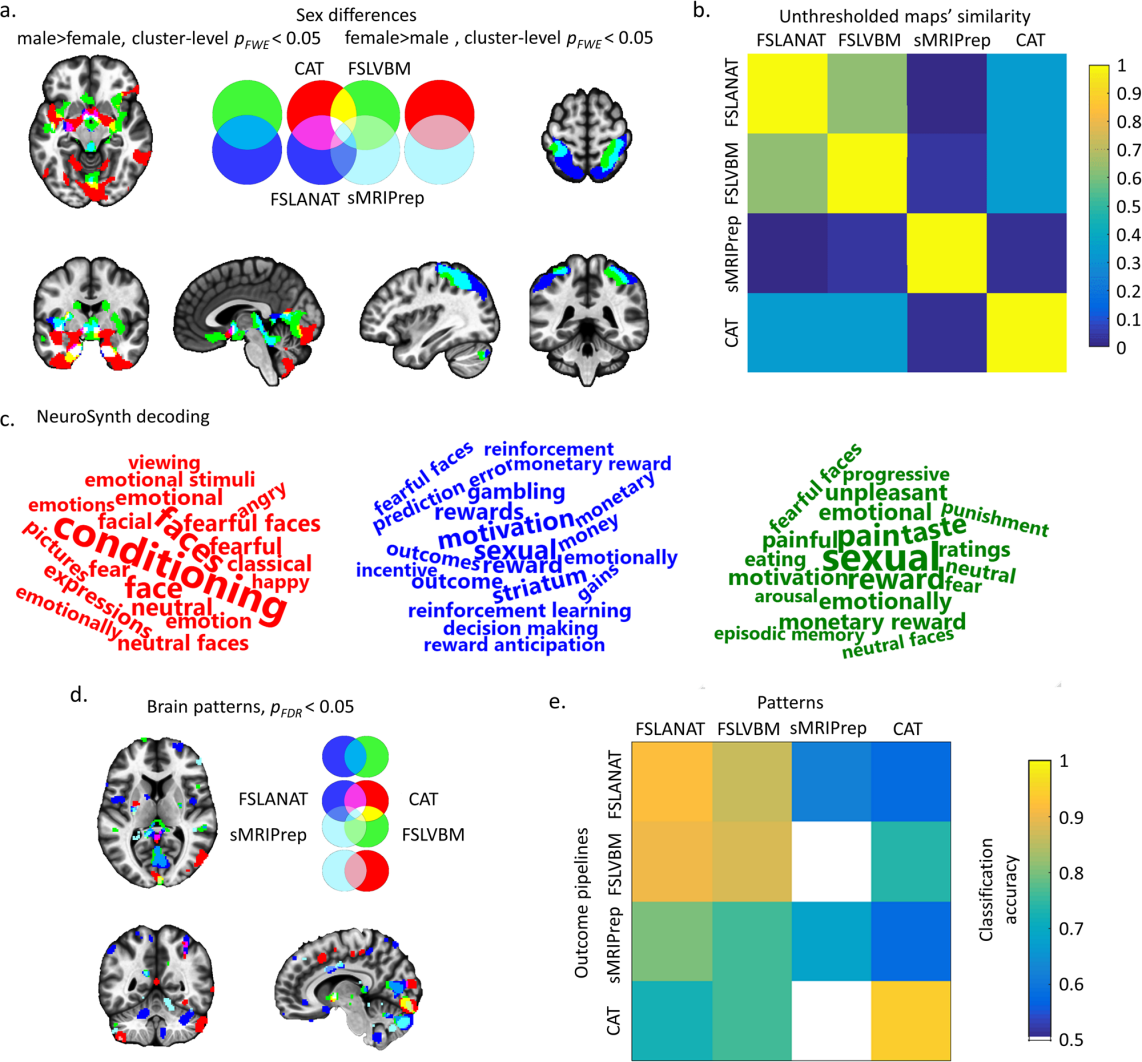
Univariate and multivariate analyses determining GMV sex differences. **a** Results from parametric statistics showing between-pipeline overlap at a cluster-level *p*_*FWE*_ < 0.05 with initial cluster forming voxel level *p* < 0.001. The left panels of **a** display results for the male>female contrast. The right panels of **a** correspond to the female > male contrast. For **a** and **d** the pipelines are coded as: red = CAT, green = FSLVBM, blue = FSLANAT, light blue = sMRIPrep, additional colors visualize the overlap between the results, e.g., CAT ∩ FSLVBM = yellow, CAT ∩ FSLANAT = purple, FSLVBM ∩ FSLANAT = light blue, CAT ∩ FSLVBM ∩ FSLANAT = white and etc. **b** The variability of unthresholded statistical maps. The correlation values between whole-brain unthresholded statistical maps of four pipelines were computed respectively for sex differences. Only positive values are visualized for display purpose. **c** Decoding the functional properties of the identified brain regions of male > female (**a**, red = CAT, green = FSLVBM, blue = FSLANAT, no difference in sMRIPrep) using NeuroSynth. Only the top 20 functional terms are visualized. The font size reflects the size of the correlation. **d** Reliable brain patterns to distinguish sex differences via bootstrapping test (5000 permutations, *p*_*FDR*_ < 0.05), and **e** cross-predicted accuracy of four *p*ipelines in independent samples. The color from cold to warm indicates increasing classification accuracy (from 0.5 to 1).

To determine the impact of the choice of processing pipeline on the results of a typical between-subject VBM study we compared male and female participants in dataset 1. To determine the extent of common and different results between the pipelines, the percentage of common and different voxels in all significant voxels across the four pipelines was calculated (Supplemental Methods). For parametric statistics with a conventional cluster-level *p*_*FWE*_ < 0.05 correction, only 10.98% spatial overlap of the results for sex differences between FSLANAT, FSLVBM, and CAT (Table 1) were observed, while the different pipelines mapped considerable pipeline-unique GMV sex-differences (up to 54.73% unique GMV sex differences identified by one, but not the other pipelines, Table 1). Notably, the data preprocessed by the sMRIPrep pipeline did not reveal significant sex differences in GMV. Between the other pipelines, overlap for male > female was observed in the lingual gyrus, precuneus, left hippocampus, bilateral parahippocampal cortex, olfactory cortex, left putamen, and left insula (Fig. 3a). No common regions for female > male were observed among the four pipelines. The two FSL pipelines shared only 13.16% overlap (Table 1), with overlapping higher GMV for females being located in the bilateral postcentral cortex, right angular, right inferior parietal lobule, and cerebellum (Fig. 3a). In contrast to the comparably small overlap between the pipelines, wide variations in the location and extent of the identified GMV sex-differences were observed specifically in medial prefrontal and occipital regions. For instance, whereas CAT revealed higher GMV in widespread cerebellar and limbic regions in men, FSLANAT and FSLVBM revealed higher GMV in widespread posterior/superior parietal regions in women (Fig. 3a).

**Table 1:** Percent overlap of GMV sex-differences as revealed by the four pipelines.

	Male > female		Female > male	
	Parametric^a^	Nonparametric^b^	Parametric	Nonparametric
CAT (unique)	54.73%	38.94%	—	—
FSLVBM (unique)	16.57%	8.14%	8.02%	2.03%
FSLANAT (unique)	1.38%	0.29%	78.82%	88.64%
sMRIPrep (unique)	—	—	—	—
CAT ∩ FSLVBM	3.76%	9.84%	—	—
CAT ∩ FSLANAT	4.55%	10.93%	—	-—
CAT ∩ sMRIPrep	—	—	—	—
FSLVBM ∩ FSLANAT	8.02%	12.97%	13.16%	9.33%
FSLVBM ∩ sMRIPrep	—	—	—	—
FSLANAT ∩ sMRIPrep	—	—	—	—
CAT ∩ FSLVBM ∩ FSLANAT	10.98%	18.89%	—	—
CAT ∩ FSLVBM ∩ sMRIPrep	—	—	—	—
CAT ∩ FSLANAT ∩ sMRIPrep	—	—-	—	—
FSLVBM ∩ FSLANAT ∩ sMRIPrep	—	—	—	—
CAT ∩ FSLVBM ∩ FSLANAT ∩ sMRIPrep	—	—	—	—

^a^Cluster-level *p*_*FWE*_ < 0.05.

^b^TFCE *p*_*FWE*_ < 0.05.

Results from the nonparametric statistics (TFCE *p*_*FWE*_ < 0.05) were highly similar to the parametric statistic results, suggesting that the pipeline differences are robust across statistical models (details, please see Supplemental Results). Notably, in some instances, the overlap between the software packages increased slightly using the nonparametric approach (Table 1 and Fig. S4a).

To further account for potential interaction effects between the preprocessing pipelines and the threshold for multiple comparisons, we computed correlations between unthresholded statistical between-group difference maps from the four pipelines with a similar approach see ref. ^34^. The spatial pattern of similarities of sex-dependent GMV differences ranged from −0.0033 to 0.6328 (Fig. 3b), with a particularly low spatial overlap of sex differences revealed by sMRIPrep compared with those obtained by other pipelines. Moreover, CAT results were very dissimilar from the sex differences obtained by the FSL pipelines.

### Meta-analytic functional characterization of the identified sex-differences

To explore the extent to which the different regions identified by the pipelines may affect the functional interpretation of brain volumetric sex-differences a meta-analytic functional decoding approach was employed (for a similar approach see ref. ^35^). The approach is based on a large-scale automated synthesis of functional MRI studies supported by platforms such as Neurosynth and is increasingly used to improve the functional characterization of a given brain region (see e.g., ref. ^36^, for conceptual background), and to aid the interpretation of GMV findings, including sex differences in GMV^35^. According to the meta-analytic decoding with Neurosynth, the identified regions between the pipelines differed strongly in terms of their functional characterization (Fig. 3c, note that only three pipelines revealed significant sex-differences). This, in turn, may have promoted quite different interpretations of potential behavioral and cognitive differences between the sexes.

### Prediction approach: sex differences in multivariate pattern analyses

MVPA-based prediction approaches have been increasingly applied to voxel-wise GMV data to determine group membership, including diagnostic groups as well as biological sex groups. To test whether the different pipelines would influence multivariate prediction accuracy, we developed pipeline-specific MVPA classifiers for sex. To this end, dataset 1 was split into a training (*n* = 100) and test (*n* = 100) dataset—each preprocessed by identical pipelines. In general, classifiers developed on each pipeline accurately predicted sex in the independent data (accuracy ranging from 68% (sMRIPrep) to 94% (CAT), Cohen’s d = 0.2967 to 2.2815, Fig. 3).

The most reliable regions for the classification of sex across pipelines encompassed the medial prefrontal, subcortical, insular, occipital, and parietal regions. Overlapping clusters of predictive voxels across pipelines were only observed in the bilateral parahippocampal cortex (voxels of each cluster > 5, Fig. 3d), and there were wide differences in the location of predictive voxels. For instance, predictions based on CAT strongly weighted voxels in the putamen, hippocampus, middle cingulate cortex, and angular gyrus, while FSLANAT identified strongly predictive voxels in a widespread network including the superior frontal cortex, orbitofrontal cortex, pre- and postcentral cortex, insula, temporal pole, angular gyrus, and cerebellum. FSLVBM and sMRIPrep revealed generally similar findings to FSLANAT.

To further validate the impact of the processing pipelines on prediction accuracy in the independent dataset, the classifiers from the training data of each pipeline were applied to the independent data processed by the other pipelines. Despite the low spatial overlap between the thresholded predictive maps (Table S11), all classifications across pipelines could accurately predict sex (58–94%, Cohen’s d = 0.1392–2.2815), with the exception of using the pattern developed on sMRIPrep to predict sex from FSLVBM processed data (50%, Cohen’s d = 0.2930) or CAT (14%, Cohen’s d = −1.4909) (Fig. 3e, corresponding Cohen’s d and performance details in Tables S12 and S13). Specifically, cross-pipeline predictions between the FSL pipelines reached the highest accuracy (>86%), as well as relatively high accuracy for predicting data processed by sMRIPrep (FSLANAT: 80%, Cohen’s d = 0.6437, and FSLVBM: 76%, Cohen’s d = 0.7231) and CAT (FSLANAT: 72%, Cohen’s d = 0.7810, and FSLVBM: 76%, Cohen’s d = 0.7402). For further independent validation of the sex-predictive pattern in dataset 2 see Supplemental Results.

### Effects of the pipeline on univariate associations: age-related effects

**Fig. 4:**
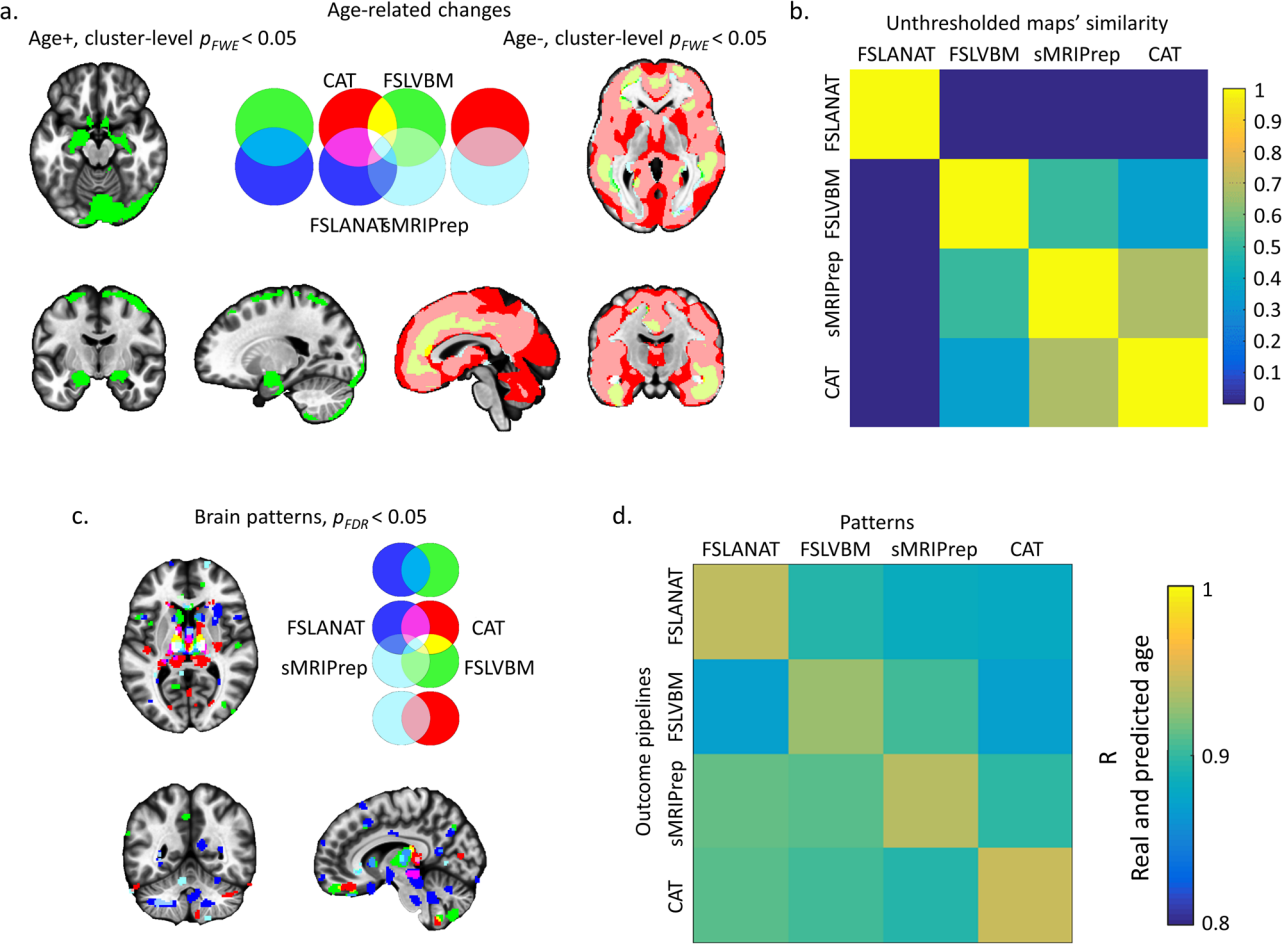
Univariate and multivariate analyses with respect to age-related GMV changes. **a** Results displaying the overlap between pipelines at a cluster-level *p*_*FWE*_ < 0.05 with initial cluster forming voxel level *p* < 0.001. The left panels of **a** depict brain regions with increasing GMV with age. The right panels of **a** depict decreases with age. For **a** and **d** the pipelines are coded as: red = CAT, green = FSLVBM, blue = FSLANAT, light blue = sMRIPrep, additional colors visualize the overlap between the results, e.g., CAT ∩ FSLVBM = yellow, CAT ∩ FSLANAT = purple, FSLVBM ∩ FSLANAT = light blue, CAT ∩ FSLVBM ∩ FSLANAT = white and etc. **b** The variability of unthresholded statistical maps. The correlation values between whole-brain unthresholded statistical maps of four pipelines were computed, respectively, for age-related effects. Only positive values are visualized for display purpose. **c** Reliable brain patterns to predict age determined by bootstrapping tests (5000 permutations, *p*_*FDR*_ < 0.05), and **d** cross-predicted r value of four pipelines for each of the pipeline proprocessed samples. The color from cold to warm indicates increasing r values (from 0.8 to 1).

In addition to determining between-group differences in brain morphometry VBM is often applied to examine associations between variations in biological variables and GMV. Associations between age and variations in GMV are, for instance, commonly interpreted in terms of age-related brain changes. To examine how the different pipelines affect the results of association studies—specifically the identification of regions that undergo age-related changes—we examined differences between the pipelines with respect to determining age-related volumetric changes in a regression approach. Using parametric statistics (cluster-level *p*_*FWE*_ < 0.05 threshold), all pipelines revealed GMV decreases with age. However, the overlap between all pipelines was only observed in the middle occipital gyrus (Fig. 4a and Table 2). Further inspection revealed that FSLANAT had rather a low overlap with the other pipelines, whereas the other three pipelines additionally identified common age-related decreases in medial prefrontal, cingulate, and some parietal and temporal regions (Fig. 4a). In contrast, age-related increases were only observed in two pipelines (FSLVBM and sMRIPrep) with minimal overlap in the cerebellum (3.41% overlap, Fig. 4a and Table 2). In general, the results showed a high variability with respect to both, the direction (FSLVBM and sMRIPrep) and the extent of the age-related effect (sMRIPrep). For nonparametric statistics with TFCE *p*_*FWE*_ < 0.05, the results were very similar to parametric statistics, particularly for brain regions that decreased with age (Fig. S4b and Table 2, details please see Supplemental Results).

**Table 2:** Percent overlap of age-associated GMV changes between the pipelines.

	Positive association		Negative association	
	Parametric^a^	Nonparametric^b^	Parametric	Nonparametric
CAT (unique)	—	0.52%	21.58%	14.35%
FSLVBM (unique)	92.6%	96.37%	1.05%	1.2%
FSLANAT (unique)	—	—	—	—
sMRIPrep (unique)	3.98%	1.87%	4.01%	4.25%
CAT ∩ FSLVBM	—	—	0.69%	0.77%
CAT ∩ FSLANAT	—	—	—	—
CAT ∩ sMRIPrep	—	—	53.77%	56.62%
FSLVBM ∩ FSLANAT	—	—	—	—
FSLVBM ∩ sMRIPrep	3.41%	1.23%	0.9%	1.05%
FSLANAT ∩ sMRIPrep	—	—	0.003%	0.003%
CAT ∩ FSLVBM ∩ FSLANAT	—	—	—	—
CAT ∩ FSLVBM ∩ sMRIPrep	—	—	17.95%	21.67%
CAT ∩ FSLANAT ∩ sMRIPrep	—	—	0.05%	0.07%
FSLVBM ∩ FSLANAT ∩ sMRIPrep	—	—	—	0.0001%
CAT ∩ FSLVBM ∩ FSLANAT ∩ sMRIPrep	—	—	0.002%	0.02%

^a^Cluster-level *p*_*FWE*_ < 0.05.

^b^TFCE *p*_*FWE*_ < 0.05.

Further examining the spatial similarity of the age-related GMV association maps by means of computing correlations between unthresholded statistical maps across four pipelines revealed variations in age-related effects ranging from −0.0051 to 0.6757 (Fig. 4b). The lowest similarity values indicated that FSLANAT produced very different maps for age-related changes as compared to the other pipelines and that CAT was rather different from the FSLVBM processing pipelines. CAT and sMRIPrep had the highest similarity with respect to the unthresholded maps. In line with the overlap results (Table 2), a considerable proportion of the variance between pipelines was introduced by the results from FSLANAT.

### Prediction approach: age-related effects from multivariate pattern analysis

Multivariate prediction models are increasingly applied to GMV maps to determine the actual age or ‘brain age’ based on brain structure. We therefore further explored the extent to which the choice of processing pipeline would affect prediction accuracy as well as the regions that contribute most to the prediction. To this end, we trained a support vector regression (SVR) model to predict age based on GMV maps. Given the lack of a suitable independent test dataset, we employed a cross-validation approach to evaluate the effects of pipelines by quantifying the correlation strengths between predicted and true age for within- and between-pipelines. With respect to the spatial overlap of pattern expressions from the four pipelines, overlapping regions were mainly located in the bilateral pallidum, bilateral thalamus, and parahippocampal gyrus (voxels of each cluster >5, Fig. 4c). Considerable spatial variations became apparent (Table S11), for instance, FSLANAT revealed high predictive weight for regions in the putamen, hippocampus, hypothalamus, brainstem, medial frontal cortex, middle temporal gyrus, middle frontal gyrus, and insula, while data processed with FSLVBM suggested that postcentral gyrus, superior frontal gyrus, superior temporal gyrus, and cerebellum strongly contributed to the prediction. Despite marked differences in the spatial distribution, an accurate prediction of age was possible based on data from all pipelines, as reflected by high correlations between the predicted and true age (all r values >0.8, Fig. 4d).

### Exploring the effects of the template and spatial similarity outliers

To address the effects of the template and quality assessment after spatial normalization, we included the following two-step approach. First, we reprocessed the data using the same brain template across pipelines. Given that the FSL-based pipelines and sMRIPrep employed study-specific templates, we employed the template from CAT across all pipelines (CAT template IXI555_MNI152). Second, we employed an assessment of sample homogeneity (inter-participant spatial similarity) within each pipeline as a quality assessment strategy and excluded images with low quality (details see Supplemental Results, Table S14). An identical strategy for quality assessment is employed by CAT, for example. Next, we implemented additional analyses to explore the effects of the template and quality assessment on between-pipeline variability in terms of sex differences and age associations, as well as a direct statistical comparison between pipelines on the voxel level.

After using the same template across pipelines, differences between sex and age effects (Fig. S5) as well as the direct statistical comparison (Figs. S6 and S7) remained stable. The variability between the pipelines remained robust after controlling for the potential effects of different TIV estimation approaches by means of aligning both template and TIV calculation across pipelines (Fig. S8). Furthermore, after controlling the template and excluding images that did not pass quality control (low spatial similarity images), results for both analyses changed considerably (Fig. S9), suggesting a strong effect of sample homogeneity. However, although between-pipeline variability could be reduced by means of template and quality control, considerable variability remained across pipelines (details see Supplemental Results, Tables S15 and S16).

## Discussion

VBM is among the most commonly employed approaches for examining regional differences or variations in brain structure in fundamental neuroscience and psychiatric neuroimaging. We here examined the variability of VBM results across commonly used software packages and pipelines. Additionally, we examined how the choice of the processing pipeline influences results in terms of the identified brain regions in two prototypical VBM study scenarios examining GMV between-group differences (sex differences) or linear associations (age-related changes). To this end, data from two independent datasets were processed with the recommended default options in widely used VBM analysis packages (CAT12, FSL, and sMRIPrep) or pipelines (FSLANAT, FSLVBM), respectively. Examining spatial similarity between the preprocessed data revealed marked differences in the voxel-level spatial distribution of GMV across the pipelines as well as with respect to the spatial homogeneity of participants’ data within the pipelines. Both, voxel-level and image-based replicability analyses revealed consistently poor replicability between pipelines, confirming considerable variations in the estimation of regional GMV. We next examined how different processing pipelines would impact the determination of GMV variations in two typical mass-univariate analytic scenarios examining between-group differences (sex differences) and associations with phenotypic variations (age associations). While some overlap in the identified regions was found across parametric and nonparametric correction procedures, there were considerable variations in both GMV sex differences and age associations, reflecting that the choice of software has a strong impact on the regions identified. In addition to mass-univariate methods, machine-learning-based approaches were applied to explore general associations between subtle spatial variations in GMV and the two biological variables of sex and age. Although the regional overlap of the most predictive voxels between the pipelines was low, GMV maps processed with each pipeline generally allowed an accurate prediction of the biological variables. Prediction accuracy varied within- and between-pipelines suggesting that the choice of processing software influences multivariate prediction accuracy. Together, the findings indicate considerable variability in the results obtained and that the choice of processing pipeline will considerably influence which regions are identified in VBM analyses. This, in turn, will strongly influence the interpretation of the findings in terms of e.g., ‘which brain regions differ between men and women’ or ‘which brain regions show age-related declines in volume’. On the other hand, the high predictive accuracy for sex and age indicates that all GMV maps encoded biologically significant variations, although region-specific interpretations need to be considered with caution.

In the first step, we examined the spatial variability and replicability of the preprocessed GMV images between the pipelines. For a biological valid and robust index that reflects regional variations in the gray matter one would expect a high spatial homogeneity as well as replicability across pipelines. However, the spatial similarity analyses revealed considerable variations between the pipelines as well as within them. Extensive variability existed both within- and between-pipelines, but notably, the samples preprocessed with CAT exhibited a higher within-participant homogeneity compared to other pipelines (Figs. S2 and S3). These findings suggest that the choice of the pipeline has a considerable influence on the spatial distribution of GMV variations and additionally influences how much individual variation is retained after preprocessing of the data. Examination of the between-pipeline replicability revealed a generally poor consistency, reflecting low inter-pipeline replicability, with the additional voxel-level replicability examination suggesting some regional variations with particularly low consistency between the pipelines in parietal and frontal cortices (Fig. 2, Table S10). In addition, high variability between the example datasets in the present study was observed (e.g., reflected by inconsistent ICC maps and pipeline performances between dataset 1 and dataset 2). This may reflect the potential influence of different acquisition protocols, MRI systems, and population characteristics, and suggests complex interactions between these factors and the specific preprocessing pipeline. Future multi-center studies and mega-analyses pooling data from different centers are required to carefully evaluate these complex interactions^33^.

Our second main aim was to examine how the choice of pipeline and implementation of the pipeline-specific default configuration would affect the results of a typical VBM study. With respect to GMV variations and biological factors, sex and age have been extensively examined in previous studies. Although the specific regions that exhibit GMV differences between men and women differ between studies, region-specific differences are commonly interpreted to underlie sex differences in cognitive and emotional functions associated with them^8–10^. Similarly, previous findings on region-specific GMV changes with age revealed inconsistent results^28,29^—even with strongly increasing sample sizes^9,10,37,38^. Age-associated changes in GMV are commonly interpreted in terms of atrophic changes that mediate specific emotional and cognitive changes with age. In contrast, the present findings indicate that the specific regions exhibiting sex differences or age-related changes strongly depend on the choice of the processing pipeline. For instance, after controlling for the influence of statistical inference (same statistical software, see also the similarity of unthresholded maps, Figs. 3 and 4) and sample or scanner differences (the same dataset was used across pipelines), only a few—or in the case of sex differences even no (Fig. 3a, Table 1)—overlapping regions were identified. Moreover, for identified GMV sex differences no three pipelines overlapped more than 20 percent, which also reflected the high regional variations. For instance, after processing with CAT, results would indicate higher GM volume in men in limbic regions typically associated with emotional processes or spatial navigation, whereas results for the FSL-based pipeline would indicate GMV sex differences in posterior parietal regions typically associated with attention or motor integration. With respect to age-related changes, the pipelines revealed some overlapping regional GMV decreases in the middle occipital gyrus, although this was generally small (0.002% and 0.02% corresponding to parametric and nonparametric statistics, respectively, Figs. 4a, S4, Table 2). In general, the location, extent, and direction of age-related GMV changes differed considerably between the pipelines. For instance, while analysis with CAT revealed widespread age-related GMV decline in nearly the entire cortex, FSLANAT revealed instead regional-specific decreases in inferior frontal regions, while FSLVBM revealed regional-specific age-related GMV increases in cerebellar and limbic regions. In line with a recent study examining the influence of pipelines on functional brain activation results^34^, we additionally examined spatial correlations between the unthresholded statistical maps. However, although this previous study reported a considerable overlap of the unthresholded functional maps^34^, cross-pipeline overlap for the GMV maps in the present study was rather low (Figs. 3 and 4), implying that the impact of pipeline additionally varies depending upon the brain modality under investigation.

In addition to mass-univariate analyses, machine-learning-based approaches were employed to investigate sex differences and age-related effects from a functional and general biological validity perspective^39,40^. Briefly, the basic idea is that some features which can be derived from the GMV maps significantly contribute to the accurate prediction of the biological variables age and sex. Notably, based on all GMV maps, reliable features for an accurate prediction of the biological variables could be extracted (e.g., for age all correlations >0.8, Fig. 4d, for sex classifiers, higher than chance level, Figs. 3e and S10). These results suggest that all pipelines retained biologically and functionally relevant information. However, further examination of the spatial distribution of the most predictive voxels revealed considerable variations across the four pipelines, similar to the mass-univariate analyses (Figs. 3d and 4c, Table S11). For instance, the application of CAT processed data to develop sex classifiers would have emphasized the region-specific contribution of the putamen, hippocampus, middle cingulate cortex, and angular gyrus, while FSLANAT would have indicated that a widely distributed pattern allowed successful sex classification. Finally, the preprocessing pipeline had a significant effect on prediction accuracy and prediction effect sizes, such that, depending on the pipeline, our sex classifiers reached 70–94% classification accuracy in an independent dataset. This indicates that the processing pipeline can have a considerable effect on the sensitivity and specificity of multivariate predictive signatures.

The impact of a number of potential sources of variability was further explored, i.e., template, TIV, and data quality effects. Our findings suggest that all of these factors contribute to the variability, but even after aligning these factors, considerable differences between the pipelines were observed. Future studies should thus consider employing standardized procedures for these factors (e.g., replication with standardized templates, TIV, and thorough data quality checks, in particular, homogeneity estimates).

The largest variations were observed between CAT and the other pipelines and remained stable after controlling some sources of variability such as template and TIV effects. The marked differences may reflect that the other three pipelines were based on FSL or incorporated FSL-based modules (the modular in-house pipeline implemented in sMRIPrep incorporated FSL functions for spatial normalization and modulation), whereas CAT employs distinct routines. For instance, CAT uses the unified segmentation from SPM for initial registration, and next optimizes segmentation with other extended options. This may promote more homogenous GMV processed images and have contributed to both, the higher homogeneity within the datasets processed by CAT as well as the marked between-pipeline differences between CAT and the other pipelines. In addition, the FSL-based pipelines create and employ study-specific templates for normalization, whereas CAT uses a prespecified template. The variability introduced by the computation of a study-specific template might further amplify the differences between the pipelines. While variability between the pipelines remained stable after employing the same template in both univariate and multivariate analyses (see supplements), we cannot rule out that differences in normalization templates may lead to variability in pathological samples with brain structural alterations. With respect to multivariate analyses we also observed variations between the pipelines; for instance, even with a constant template the sMRIPrep pipeline data yielded only a comparably low predictive accuracy which may reflect low internal consistency in dataset 1 when processed by the sMRIPrep pipeline (see Fig. S3). Finally, CAT and FSL recommend different strategies to modulate data, i.e., affine + nonlinear and nonlinear only modulation, which may have led to variability between the pipelines. The exact influence of the modulation has been examined in previous studies for within-pipeline scenarios see e.g., refs. ^41,42^.

Our findings challenge the reproducibility as well as biological and functional interpretability of regional GMV variations as determined by VBM. The choice of software had a considerable impact on the regional variation of GMV on the voxel level, which is difficult to reconcile with a biologically valid index. Moreover, regions that were found to exhibit sex differences or age-related GMV changes differed strongly depending on the pipeline employed. The high variability in regions identified would have led to a rather different functional interpretation of sex differences (e.g., Fig. 3c) as well as atrophic changes with age and potential functional consequences. In contrast, multivariate analyses accurately predicted age and gender with classifiers trained on GMV maps from all pipelines; however, the specific predictive regions differed. Together, these findings indicate that GMV indices encode biologically relevant information, yet the interpretation of specific regions in both univariate, as well as multivariate analyses, will, to a large extent, be pipeline-dependent. In the context of the replicability crisis, meta-analyses of neuroimaging data are considered the gold standard, but our current findings indicate that coordinate-based meta-analyses may also need to account for regional variability between studies introduced by the use of different pipelines.

The present findings emphasize the need for detailed reporting of the software specifications and configurations, which is also advocated by the Committee on Best Practices in Data Analysis and Sharing (COBIDAS) report^43^. However, the fact that the pipelines with recommended default configurations revealed considerably different GMV results indicates that further efforts are needed to promote the development of robust and reproducible GMV-based biomarkers^34,44,45^. Potential initial steps are open cooperation and replicability analyses across software platforms and pipelines, open software platforms that allow comparisons and standardization of methods across platforms, and a transparent and detailed processing report that should accompany manuscript submissions (e.g., as provided by sMRIPrep and fMRIPrep). Further analyses exploring the effects of normalization template, TIV calculation, and data quality in terms of sample homogeneity revealed that between-pipeline variability remained robust when aligning template and TIV across pipelines. In contrast, quality control of the preprocessed images by means of excluding images with low spatial similarity reduced between-pipeline variability to some extent. These findings imply that improving data homogeneity may improve replicability across pipelines. However, depending on the pipeline, different images were excluded based on the sample homogeneity. Moreover, the lack of a gold standard for analytic flexibility in neuroimaging meta-analysis and the lack of ground truth for VBM indices limits comparison between pipelines. For instance, pipelines may exhibit high within-pipeline replicability; however, the current work does not allow us to specify which of the identified regions represent true positive results. Despite this limitation, the present work demonstrates that the choice of a specific VBM pipeline will strongly influence the results obtained for the same research question. To further determine true positives and the biological plausibility of the VBM technique will require benchmarking with clear biological indices from animal models, postmortem brain indices, or invasive approaches. Finally, although the present study mainly focuses on the initial examination of variability introduced by the different VBM pipelines, a number of recent studies examined within-pipeline reliability for brain structural measures, including VBM, across different timepoints^46–49^. These studies generally reported good to excellent within-pipeline VBM reliability, although it was influenced by participant characteristics such as sex or the presence of a disorder. A recent study examined between-pipeline variability for functional MRI and employed a densely sampled test-retest dataset for evaluation^50^, an approach that would allow the identification of interactions between-pipeline variability and repeated assessments in future studies on VBM. In addition, although the different software packages refer to general preprocessing steps such as “segmentation” or “spatial normalization”, details in the implementation may lead to highly variable results. It will be necessary to explore automatic quality control tools and establish an overarching and modular workflow to enhance robustness of VBM analyses.

The present study demonstrated considerable variations in GMV indices and corresponding results across the most commonly used processing pipelines for VBM. The combination of mass-univariate analyses and machine-learning-based multivariate approaches revealed that the specific regions identified to exhibit GMV sex-differences or age-related changes varied strongly depending on the software chosen. While multivariate prediction of sex and age was possible across pipelines, prediction accuracy varied strongly between them. Together, these findings challenge the interpretability and robustness of VBM results.

## Methods

### Datasets

Dataset 1 included T1-weighted anatomical data from 200 healthy Chinese participants aged 18–26 years old (mean = 21.45 years old, SD = 2.18; 100 females and 100 males matched for age; sample details see also Liu, et al.^51^). This dataset served to determine variations across the four analytic pipelines with respect to determining gray matter differences in between-subject designs using the example of sex differences.

Dataset 2 included 494 healthy Chinese participants aged 19–80 years (mean = 45.18 years, SD = 17.44, 187 males) from an openly available dataset (SALD) encompassing T1-weighted anatomical and resting-state functional MRI data (details please see ref. ^52^). This dataset served to determine variations between the software packages with respect to linear associations between biological indices and GMV with the example of age-related changes. For detailed structural MRI acquisition parameters, please see Supplemental Methods.

### Data quality control

First, we inspected apparent artifacts and image quality by visual inspection, which confirmed high image quality. Second, automated quality assessment by the MRIQC toolbox (https://mriqc.readthedocs.io/)^53^ was employed to further evaluate raw data quality, including signal-to-noise ratio (SNR), foreground to background energy ratio (FBER), percent of artefact voxels (Qi1) (details and results see Supplemental Material Figs. S11 and S12 and Wei et al.^52^). Third, the CAT12.7 (r1720) (http://www.neuro.uni-jena.de/cat/) quality assurance (QA) framework for empirical quantification of quality differences across scans and studies were applied. This retrospective QA allows the evaluation of essential image parameters such as noise, inhomogeneities, and image resolution which can be integrated into a single quality index (dataset 1: mean = 81.68, SD = 1.61, range = 73.48–84.48; dataset 2: mean = 84.51, SD = 1.27, range = 76.9–86.24; scores >70 indicates satisfactory to excellent image quality). Thus, all data passed the quality control procedure.

### Preprocessing pipelines

VBM analyses commonly write out two types of structural indices, referred to as volume and concentration, depending on whether a modulation step is employed or not^37,54,55^. In line with the advantages of, and wider use of, modulated images (volume), all subsequent analyses focused on modulated data.

With respect to the preprocessing pipelines we established four separate preprocessing pipelines. These pipelines implemented a voxel-wise estimation of local GMV. Given that the primary aim of our study was to examine variability introduced by the use of commonly used VBM pipelines, the four pipelines were set up according to the default or recommended configurations in the respective manuals of the software packages (Fig. 1). One pipeline was based on CAT12.7 (r1720) (http://www.neuro.uni-jena.de/cat/) (CAT); two pipelines were based on FSL v6.0 (https://fsl.fmrib.ox.ac.uk/fsl/fslwiki/FSL, Smith et al.^56^, Jenkinson et al.^57^) (FSLVBM and FSLANAT, respectively); and one pipeline included modules from different software packages and was based on sMRIPrep 0.6.2 (Esteban et al.^58^, RRID:SCR_016216, https://www.nipreps.org/smriprep/) (sMRIPrep).

The CAT pipeline was implemented in CAT12.7 running on SPM12 v7219 (Welcome Department of Cognitive Neurology, London, UK, https://www.fil.ion.ucl.ac.uk/spm/software/spm12/). Standard VBM preprocessing protocols of CAT12 as outlined in the CAT12.7 manual were employed in the pipeline. Briefly, the T1-weighted images were bias-corrected, segmented into gray matter (GM), white matter (WM), and cerebrospinal fluid (CSF) using SPM’s unified segmentation function for segmentation and initial registration, with additional optimization of the segmentation (e.g. using local adaptive segmentation and adaptive maximum a posterior segmentation) and spatially normalized to the standard Montreal Neurological Institute (MNI) space using the ICBM-152 template (East Asian, additional results obtained with the Caucasian template did not affect the results, see supplements Fig. S13) with a voxel size of 2 × 2 × 2 mm. GM images were smoothed with three Gaussian kernels with commonly used smoothing kernels (8, 10, and 12 mm) at full width at half maximum (FWHM) for subsequent statistical analysis and total intracranial volume (TIV) was estimated to correct for individual differences in brain size. Default parameters were applied unless indicated otherwise.

Two different default preprocessing pipelines were established in FSL^56,57^: (1) FSLVBM (https://fsl.fmrib.ox.ac.uk/fsl/fslwiki/FSLVBM), and (2) FSLANAT (https://fsl.fmrib.ox.ac.uk/fsl/fslwiki/fsl_anat). The FSLVBM default pipeline included the following four steps: First, non-brain tissue was removed using BET (fslvbm_1_bet). Second (fslvbm_2_template), tissue-type segmentation was conducted via the Automated Segmentation Tool (FAST), to segment the images into GM, WM, and CSF. Third, the outcomes were non-linearly registered to the GM ICBM-152 template using the registration tool FNIRT, then creating a study-specific template. Finally, the GM images were non-linearly registered to the study-specific template using FNIRT (fslvbm_3_proc). In contrast, FSLANAT is a general pipeline for processing anatomical images encompassing the following steps (fsl_anat). Of note, the processing order is different from FSLVBM, and the final outcomes are segmented data in the native space. First, all T1-weighted images were reorientated to the standard MNI orientation (fslreorient2std) and automatically cropped (robustfov). Second, bias-field correction for RF/B1-inhomogeneity-correction (FAST) was done. Third, the pipeline did brain-extraction (BET) and tissue-type segmentation (FAST). The calculation of TIV for both FSLANAT and FSLVBM adhered to the protocols provided by the ENIGMA project (http://enigma.ini.usc.edu/protocols/imaging-protocols/protocol-for-brain-and-intracranial-volumes/#fsl).

sMRIPrep 0.6.2 (Esteban, et al.^58^, RRID:SCR_016216, https://www.nipreps.org/smriprep/) is a structural MRI data preprocessing pipeline designed to provide an easily accessible, state-of-the-art interface that is robust to variations in scan acquisition protocols and that requires minimal user input, while providing easily interpretable and comprehensive error and output reporting. The workflow is based on Nipype 1.5.0 (Gorgolewski, et al.^59^, RRID:SCR_002502). A similar workflow is also used in fMRIPrep anatomical preprocessing workflow (Esteban, et al.^58^, https://fmriprep.org/). In the present study, the T1-weighted (T1w) image was corrected for intensity non-uniformity (INU) with N4BiasFieldCorrection^60^, distributed with ANTs 2.2.0 (Avants et al.^61^, RRID:SCR_004757), and used as T1w-reference throughout the workflow. The T1w-reference was then skull-stripped with a Nipype implementation of the antsBrainExtraction.sh workflow (from ANTs), using OASIS30ANTs as the target template. Brain tissue segmentation of CSF, WM, and GM was performed on the brain-extracted T1w using fast (FSL 5.0.9, RRID:SCR_002823, Zhang et al.^62^). Considering no TIV estimation is provided by sMRIPrep, the corresponding brain size for the analysis was computed by summarizing the tissue types (GM + WM + CSF).

To reduce further variability induced by spatial normalization the FSLANAT, FSLVBM, and sMRIPrep pipelines used the same normalization. In detail, sMRIPrep and FSLANAT were mainly used for segmenting GM, WM, and CSF data, and the data next was integrated into the FSLVBM pipeline. For the processing in these pipelines we thus excluded the initial brain-extraction (fslvbm_1_bet) and segmentation (first part of fslvbm_2_template) stages and subjected the segmented GM outcomes (in native space) to fslvbm_2_template and fslvbm_3_proc to produce modulated GM data.

To keep preprocessing consistent within each platform the fslmaths function was used to smooth FSL processed data (FSLVBM, FSLANAT, and sMRIPrep) with comparable smoothing kernels (sigma = 3.5, 4.3, 5.2, approximately corresponding to FWHM—3.5 × 2.3 = 8.05 ≈ 8, 4.3 × 2.3 = 9.89 ≈ 10, and 5.2 × 2.3 = 11.96 ≈ 12) as the CAT data. For the CAT preprocessing, SPM smoothing was conducted with FWHM = 8, 10, and 12, respectively.

In summarizing, according to the functions used to process the data, the four pipelines could be divided into three FSL-based (FSLANAT, FSLVBM, and sMRIPrep which employ the segmentation, FAST, function from FSL) and on CAT-based pipelines. Within the FSL-based pipelines the FSLVBM employed further processing after segmentation while FSLANAT employed a different order of the processing steps. The sMRIPrep pipeline represents an in-house pipeline based on different neuroimaging packages, including FSL, Nipype, ANTs and etc. CAT employed functionally similar steps such as bias correction, segmentation, spatial normalization, smoothing, and modulation, yet these steps were based on CAT-specific rather than FSL-based processing approaches. Moreover, CAT incorporates additional steps such as the unified segmentation implemented by SPM12, and further optimization steps such as denoising. The different processing steps and functions may introduce variability in data preprocessing and statistical results. Of note, our main focus was to determine differences that can result from the application of the default or recommended processing steps within the different pipelines rather than specifically segregating the technical details that lead to the variability.

### Spatial similarity

Pearson’s correlation coefficients were employed to compute the spatial similarity of the modulated GM maps of the preprocessed data from the four pipelines for dataset 1 (male and female) and dataset 2, and at different smoothing kernels (see Fig. S2). Spatial similarity maps across the processing pipelines were established to show its distribution (Fig. S2) and revealed highly similar patterns across the unsmoothed data and data processed with three different smoothing kernels (FWHM 8, 10, and 12). Further statistical analyses therefore focused on the z-transformed r values of the FWHM 8 smoothed data. Examination of the spatial similarity within-pipeline similarities by means of ANOVA models revealed a significant main effect of pipeline, in particular, a high spatial similarity within the data processed by the CAT pipeline and a high variation between pipelines (Fig. S3, unsmoothed data see Fig. S14) for both dataset 1 and 2 (Bonferroni corrected *p* < 0.01).

### Replicability

Replicability across pipelines was evaluated using two approaches: (1) voxel-level univariate replicability was examined using the intraclass correlation coefficient (ICC) implemented by a linear mixed model in DPABI^63^, see supplemental material, and (2) whole-brain multivariate replicability, using the image intraclass correlation coefficient (I2C2), which represents a multivariate image measurement error model^5^. ICC (ICC (3,1) with linear mixed models as used in the current study) and I2C2 can estimate the consistency between the different pipelines on the voxel or whole-brain level, respectively^5,64^. The replicability between the pipelines as assessed by the coefficient is commonly interpreted as follows: <0.4 poor; 0.4–0.59 fair; 0.60–0.74 good; >0.74 excellent^64–66^.

### Univariate analyses

To account for potential interactions between preprocessing and inferential statistical procedures, all univariate analyses in the current study were conducted in SPM12 and across different multiple comparisons corrections. The analyses including conventional statistical parameter tests (threshold at voxel level *p* < 0.001, and cluster-level *p*_*FWE*_ < 0.05 with initial cluster forming voxel level *p* < 0.001 respectively) as well as threshold-free cluster enhancement (TFCE with 5,000 permutations, threshold at *p* < 0.001, and *p*_*FWE*_ < 0.05, respectively). For completeness, the uncorrected voxel-level results (*p* < 0.001 and TFCE *p* < 0.001) are provided in Supplemental Material Figs. S15 and S16, and results after excluding template effect and TIV calculation in Fig. S17.

### Between-group difference approach: sex differences in univariate analyses

Independent sample t-tests were employed to determine significant differences in regional gray matter volume between men and women. Age and TIV were included in the models as recommended for VBM analyses to control for age- and global brain size-related variations.

### Association approach: age-related changes in univariate analyses

Multiple linear regression models were employed to explore associations between age and regional GMV, including sex and TIV as covariates.

### Multivariate pattern analysis approach: prediction of sex and age

State of the art machine-learning framework in neuroimaging^40^ was adopted to explore whether the use of the different pipelines will affect the prediction accuracy of sex and age by means of distributed brain structural variations (GMV maps). For the categorical prediction (sex), the 200 healthy participants from dataset 1 were divided into two sex- and age-matched independent samples, which served as training and test datasets, respectively. A support vector machine (SVM, C = 1) was employed to develop an MVPA-based sex classifier. The SVM was trained on the training data (*n* = 100) with a bootstrapping test to find stable features (5000 permutations, pFDR < 0.05). Next, these features were used to train the model by means of five-fold cross-validation. The resulting patterns were subsequently tested in the independent test sample (*n* = 100) to determine within- and between-pipeline prediction accuracy for sex. To estimate the effect size of each classification Cohen’s d for between-subject designs was employed^67^. For prediction of a continuous variable (age) a support vector regression (SVR, epsilon = 0.1, C = 1) model was trained on dataset 2. A bootstrapping test (5000 permutations, pFDR < 0.05) was used to find stable features. These features and a five-fold cross-validation were applied to train the model. Prediction performance was next quantified by evaluation of correlation strengths between predicted and true age for within- and between-pipelines. Stable predicted performance after excluding template effect and stable predicted patterns were also provided in Figs. S18 and S19.

Of note, the aim of the MVPA was not to determine an optimized algorithm or feature set to predict sex or age but rather to determine whether different processing pipelines affect prediction accuracy and whether the GMV maps generally encode biologically meaningful information.

### Reporting summary

Further information on research design is available in the Nature Research Reporting Summary linked to this article.

## Supplementary information

Supplementary Information

Reporting Summary

## Data Availability

Unthresholded statistical maps and pattern weight images are available onOSF (https://osf.io/p5b6f/). Dataset 2 is available for download in an Amazon Web Services S3 bucket from the International Data-sharing Initiative (http://fcon_1000.projects.nitrc.org/indi/s3/index.html) under a Creative Commons License: Attribution Non-Commercial. Other data can be obtained from the corresponding authors upon reasonable request.
