# Supplementary material for: Choice of Voxel-based Morphometry processing pipeline drives variability in the location of neuroanatomical brain markers
Source: Commun Biol. 2022 Sep 6;5:913. doi: 10.1038/s42003-022-03880-1 (PMC9448776; doi:10.1038/s42003-022-03880-1)
Supplement: Supplementary file 1 — Supplementary Information [file 42003_2022_3880_MOESM1_ESM.pdf]

Supplemental material

Zhou et al.

Supplemental Figures S1-S19
Supplemental Tables S1-S16

Correspondence:
Benjamin Becker, (ben_becker@gmx.de)
Xinqi Zhou, (xinqizhou.uestc@outlook.com)
Center for Information in Medicine
University of Electronic Science and Technology of China
Chengdu 611731, China
Tel.: +86 2861 830 811

Supplemental methods

Structural MRI acquisition parameters

Both datasets were acquired using validated T1-weighted brain structural acquisition protocols. Dataset 1 was acquired on a 3.0 Tesla GE MR750 system (General Electric Medical Systems, Milwaukee, WI, USA). T1-weighted high-resolution anatomical images were acquired with a spoiled gradient echo pulse sequence, repetition time (TR) = 5.9 ms, echo time (TE) = 2 ms, flip angle = 9°, field of view (FOV) = 256 × 256 mm, acquisition matrix = 256 × 256, thickness = 1 mm, number of slices = 156, voxel size=1×1×1 mm. Dataset 2 was collected using a 3.0-T Siemens Trio MRI scanner (Siemens Medical, Erlangen, Germany). A magnetization-prepared rapid gradient echo (MPRAGE) sequence was used to acquire high-resolution T1-weighted anatomical images (repetition time = 1,900 ms, echo time = 2.52 ms, inversion time = 900 ms, flip angle = 90°, resolution matrix = 256 × 256, slices = 176, thickness = 1.0 mm, and voxel size = 1×1×1 mm)[1].

Overlapping percent of mass-univariate analyses

To estimate the consistency in terms of spatial overlap between the pipelines percent overlapping voxels were calculated for the pipeline-specific results on sex differences and age-related changes respectively (Table S10 & S11). The following formula was applied:

$$p_i = \frac{v_i}{v_{all}} \times 100$$

p_i indicates the overlapping percent of ith overlapping or independent cluster voxels (v_i) in voxels of all significant results (v_{all}), which represents the union set of the four pipelines.

ICC calculation

To estimate the replicability of preprocessed data across pipelines the intraclass correlation coefficient (ICC) implemented by a linear mixed model in DPABI was used[2]. In DPABI two forms of voxel-wise ICC calculation are provided. The current study employed ICC(3,1) using linear mixed models, which means each target is assessed by the same raters and these raters are the only raters of interest (i.e. pipelines in our study). As described in the previous literature[2] a two-level linear mixed model was applied to the decomposition of Yij (the value of the j-th participant's i-th measurement occasion). In the current case, Yij denotes the GM from the j-th participant's i-th pipeline. The two-level linear mixed model was applied to each voxel as the following decomposition of Yij:

$$Y_{ij} = \mu_j + e_{ij}$$
$$\mu_j = \mu' + e'_j$$

where μ' is a fixed parameter and e_{ij} and e'_j are independent random effects which have normal distribution with mean 0 and variances δ_e^2 and $\delta_{e'}^2$. The term e'_j is the participant effect and e_{ij} is the measurement error across pipelines. The

variance terms are estimated with the restricted maximum likelihood approach. Thus the ICC formula is defined as:

$$ICC = \frac{\delta_{e'}^2}{\delta_{e'}^2 + \delta_e^2}$$

Supplemental results

Spatial similarity within- and between-pipelines

In the male sample, the ANOVA revealed that main effects of pipeline were significant with respect to all pipeline and sample homogeneity comparisons, including between pipelines and between participants (F = 4635, p < 0.0001), between pipelines and within participants (F = 179, p < 0.0001), and within pipelines and between participants (F = 14208, p < 0.0001). The post hoc tests were conducted with appropriate Bonferroni correction for the number of tests (Table S1, S3, and S5).

In the female sample, the ANOVA revealed that main effects of pipeline were significant with respect to all pipeline and sample homogeneity comparisons, including between pipelines and between participants (F = 3535, p <0.0001), between pipelines and within participants (F = 196.1, p <0.0001), and within pipelines and between participants (F = 9894, p <0.0001). The post hoc tests were conducted with appropriate Bonferroni correction for the number of tests (Table S2, S4, and S6).

For dataset 2, the ANOVA revealed that main effects of pipeline for all homogeneity comparisons, including between pipelines and between participants (F = 61376, p <0.0001), between pipelines and within participants (F = 593.7, p <0.0001), and within pipelines and between participants (F = 290745, p <0.0001). Post hoc tests were conducted with Bonferroni's correction for the number of tests (Table S7, S8, and S9).

In summary, the spatial similarity analyses for three homogeneity comparisons revealed significant main effects of pipeline, in particular a high spatial similarity within the data processed by the CAT pipeline and a high variation between pipelines (Fig. S2 and S3) for both dataset 1 and 2 (Bonferroni corrected p < 0.01).

Between-group approach: sex differences univariate analyses

Results from the non-parametric statistics with TFCE p_{FWE} < 0.05 were highly similar to the parametric statistic results, suggesting that the pipeline differences are robust across statistic models. For instance, across pipelines males had higher GMV than females (FSLANAT, FSLVBM, and CAT had 18.89% overlaps, Table 1) in the precuneus, bilateral putamen, insula, olfactory cortex, parahippocampal cortex, and cerebellum (Fig.S4a, while for the FSL pipelines (FSLANAT and FSLVBM had 9.33% overlap, Table 1) females had higher GMV in inferior parietal lobule, postcentral cortex, and angular gyrus. Again, the software packages revealed widespread differences with respect to sex-differences in GMV in limbic, frontal and cerebellar regions. Notably, in some instances the overlap between the software packages increased slightly using the non-parametric approach (Table 1 and Fig. S4a).

Association approach: age-related effects from univariate analyses

Regarding to non-parametric statistics with TFCE $p_{FWE} < 0.05$, the results were very similar with parametric statistics, especially for the brain regions that decreased with age. FSLVBM revealed age-related increases from prefrontal cortex to parietal lobe to cerebellum, and bilateral hippocampus, while sMRIPrep revealed caudate and cerebellum. In addition, CAT highlighted thalamus, but only FSLVBM and sMRIPrep identified common cerebellar regions that increased with age (Fig. S4b and Table 2). Whereas CAT and sMRIPrep revealed age-related decreases in widespread regions covering nearly the entire cortex, the other pipelines revealed more regional-specific decreases with age, such that FSLANAT revealed specific decreases in the inferior frontal gyrus and middle occipital gyrus. FSLVBM additionally revealed regional decreases in middle cingulate cortex, frontal and temporal cortex. Again, the common brain regions across four pipelines that decreased with age only included the middle occipital gyrus (Fig. S4b). Except for FSLANAT the common regions of the other pipelines included medial prefrontal cortex, cingulate gyrus, precuneus, temporal lobe, parietal lobe, middle occipital gyrus, insula, and cerebellum (Fig. S4b).

Validation of the multivariate sex-predictive pattern in dataset 2

The developed sex-predictive patterns were further validated on dataset 2 (Fig. S10) with an averaged classification accuracy of 66.33% (SD = 3.22, range = 61.94% ~ 71.86%). Given that the age range in dataset 2 was considerably higher than in the initial training dataset we limited the age range in dataset 2 to <=30 years, which increased classification accuracy in this sample (n = 159, female = 99) to an average of 71.82% (SD = 6.33, range = 61.64% ~ 87.42%, corresponding Cohen's d in Table S10). The highest accuracy (87.42%, Cohen's d = 2.2062) appeared when using the pattern from CAT on data processed by CAT pipeline, followed by the pattern developed from FSLANAT applied to data processed by sMRIPrep (77.99%, Cohen's d = 1.2160).

Exploring the effects of template and spatial similarity outliers

To address the effects of template across all pipelines we did additional analyses with all data processed by a common template from CAT. Of note this step can explore the template differences, yet given that the spatial registration algorithms differ, i.e. geodesic shooting registration in CAT[3] vs non-linear registration in the other pipelines (https://fsl.fmrib.ox.ac.uk/fsl/fslwiki/FNIRT/UserGuide), this approach can only help to determine template effects per se but not differences due to registration algorithm or template times registration interaction effects. First we explored the sex differences and age associations as same as the main manuscript. Then the results generally confirmed that differences between pipelines remained after controlling for the different template (Fig. S5). Second we conducted a direct statistical comparison between the pipeline data via a within-subject one-way ANOVA with sex, age and TIV for original preprocessed data, new preprocessed data with a common template and

the new preprocessed data plus same TIV calculation respectively. The results from the ANOVA as well as post-hoc comparisons directly comparing the pipelines further confirmed that significant and widespread differences between pipelines could be observed not only in the processing according to the pipeline manuals but also after processing with same template, or processing with same template and identical TIV calculation (Fig. S6 & S7 & S8), respectively.

To address the quality assessment after spatial normalization with a common template, we checked the images by means of assessing sample homogeneity (inter-participant spatial similarity) a quality assessment approach that is also employed by CAT. Although, the relationship between sample homogeneity and image quality is not clear, looking for outliers in a population of subjects (or images in this case) represents a basic but robust quality assessment procedure. To this end we first calculated the inter-participant correlation matrix within each pre-processed dataset and pipeline. Next, images with a mean correlation below 2 standard deviations were identified within each dataset leading to the exclusion of different subjects: dataset 1: n = 7 for CAT, n = 11 for FSLVBM, n = 7 for FSLANAT, n = 10 for sMRIPrep, dataset 2: n = 23 for FSLVBM, n = 8 for FSLANAT, n = 14 for sMRIPrep, n = 22 for CAT. Next we reran the main analyses assessing effects of pipeline on sex-differences and age associations. Briefly, the results revealed that after controlling template effect and excluding low spatial similarity images results for both analyses changed (Fig. S9). In particular, for the sex-differences only the FSLVBM pipeline showed significant differences for both male > female and male < female in the dataset 1, while in dataset 2 results of positive age association remained stable yet for the negative association a considerable increase in overlap between the pipelines was observed (Table S14, 48.60% compared to 0.002%, see also Table 2 in the main text).

Additional exploratory MVPA analysis for sex differences

To explore whether integrating all features from all pipelines would enhance the predicted performance we concatenated the training data from each pipeline and we detected stable features (GMV) with bootstrapping test (5,000, $p_{FDR}<0.05$) for sex prediction. The identified brain pattern considerably overlapped with the patterns determined by each pipeline (Fig. S19a). Then this pattern was used to predict independent test data which was concatenated too (Fig. S19b), and independent data from each pipeline (Fig. S19c). In general, the cross-pipeline prediction improved while the within-pipeline prediction slightly decreased. Together this may tentatively suggest that utilizing data processed by different pipelines may improve the performance and generalization of MVPA-based GMV map decoders which opens interesting opportunities to improve biomarker development.

Supplemental figures

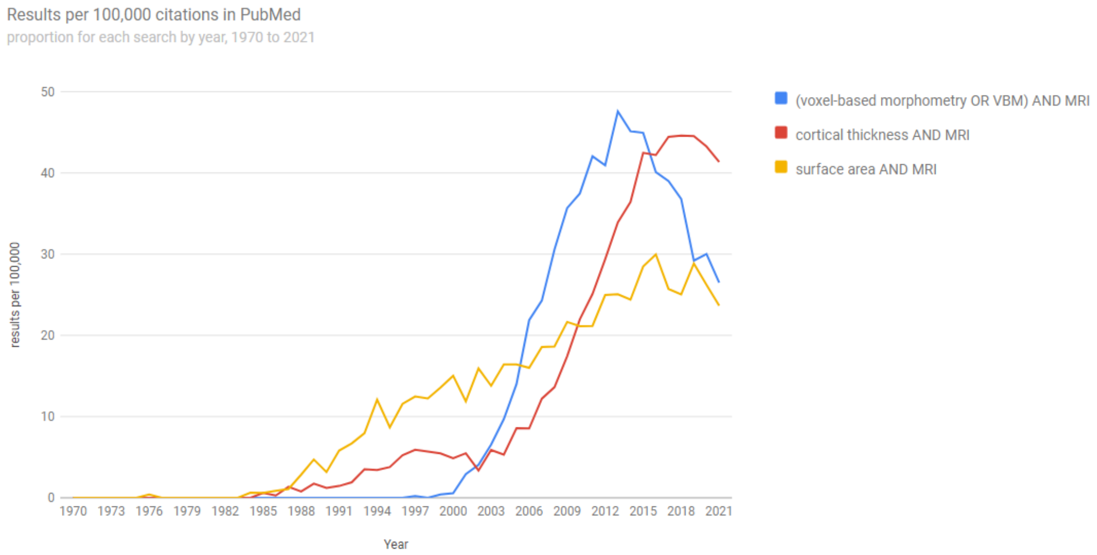

Fig. S1. Results per 100,000 citations of searching terms "(voxel-based morphometry OR VBM) AND MRI", "cortical thickness AND MRI", and "surface area AND MRI" in PubMed created by https://esperr.github.io/pubmed-by-year.

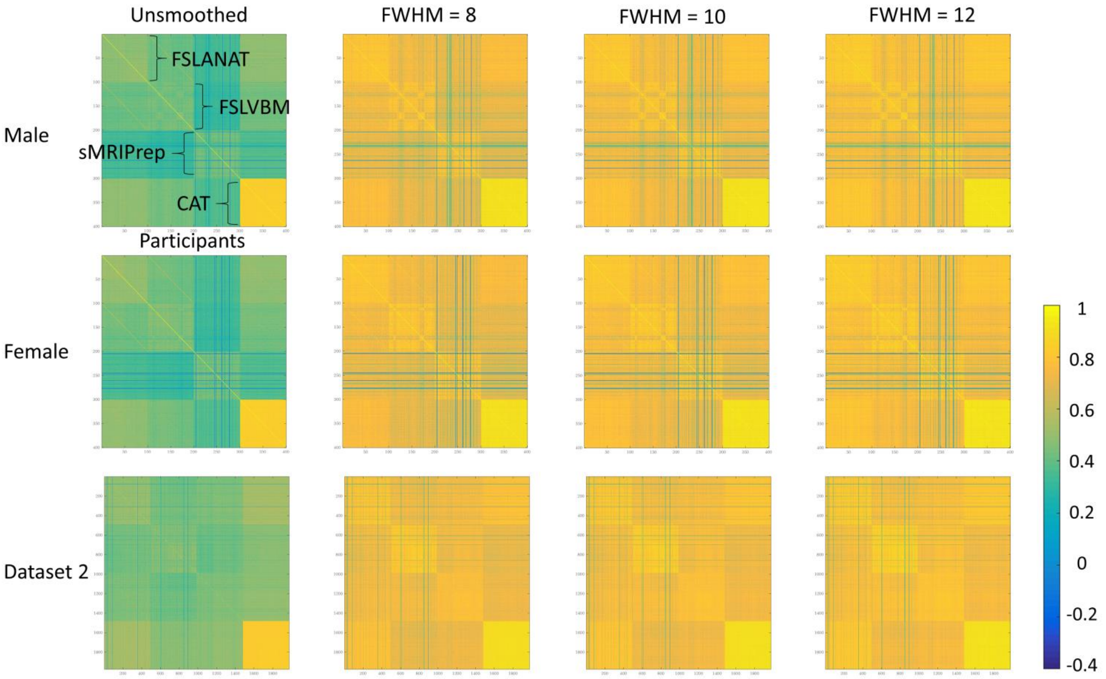

Fig. S2. Spatial similar maps across the processing pipelines. Top and middle rows correspond to male and female samples separately from dataset 1. The bottom row shows data from the entire sample of dataset 2. Each column corresponds to a smoothing level (unsmoothed, 8, 10, and 12mm FWHM). The color grading reflect r values ranging from -0.4 to 1 (no r value was lower than -0.4). Each line of both x and y axes in each matric map refers to one participant.

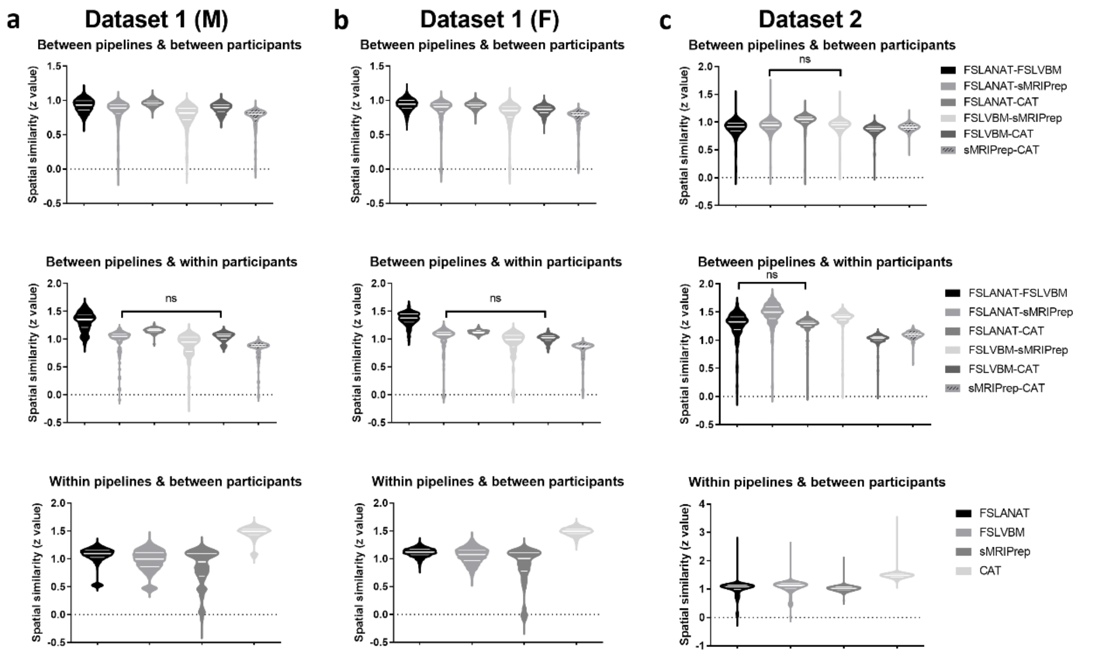

Fig. S3. Mean similarity and standard deviation (SD) of different pipelines and pipeline pairs. Column a and b display the data from dataset 1 for males (a) and females (b) respectively. Column c displays the data from dataset 2. Post hoc tests were controlled for multiple comparison using a Bonferroni corrected p < 0.01. M = males, F = females, n.s. = non-significant.

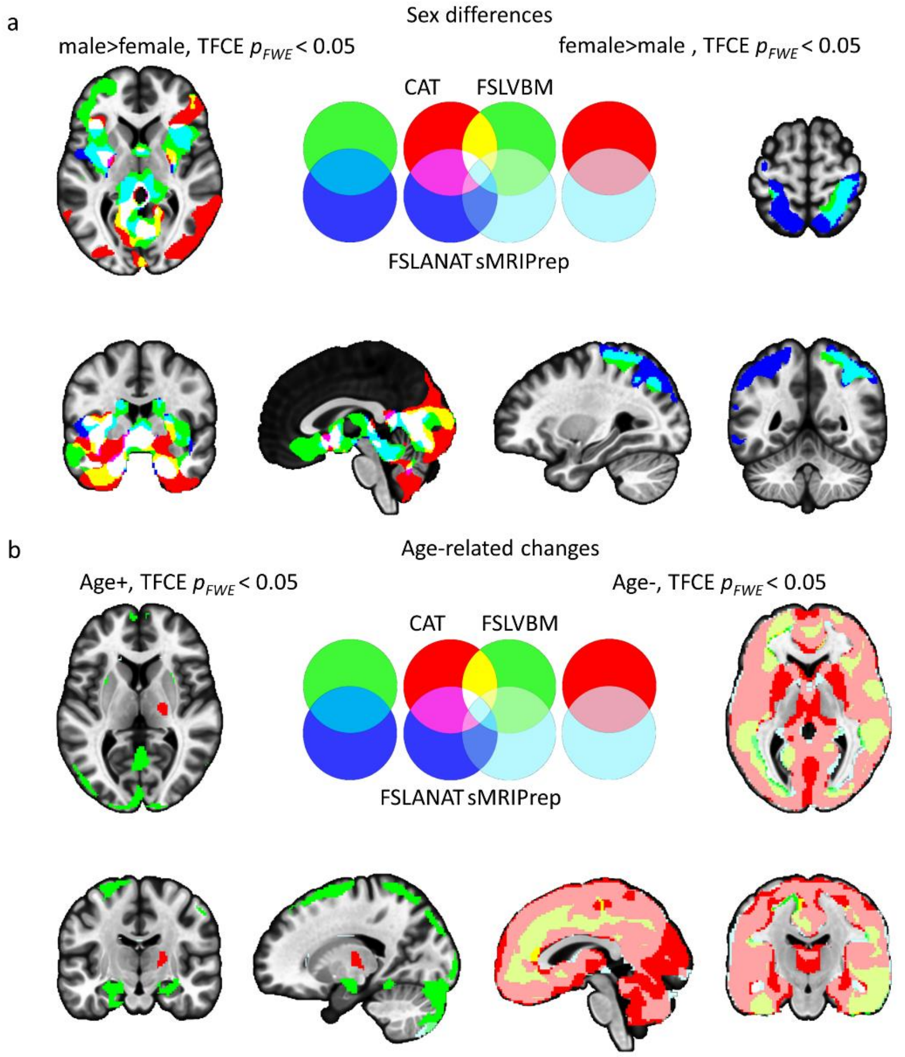

Fig. S4. Similarities and dissimilarities between the pipelines with respect to determining GMV sex differences. Displayed of a and b are results from non-parametric statistics (TFCE with 5,000 permutations) overlapping at p_{FWE} < 0.05. The left panels of a display results for the male>female contrast. The right panels of a correspond to the female>male contrast. The left panels of b depicts brain regions with increasing GMV with age. The right panels of b depict decreases with age. Red = CAT, green = FSLVBM, blue = FSLANAT, light blue = sMRIPrep, other colors visualize the overlap between the results.

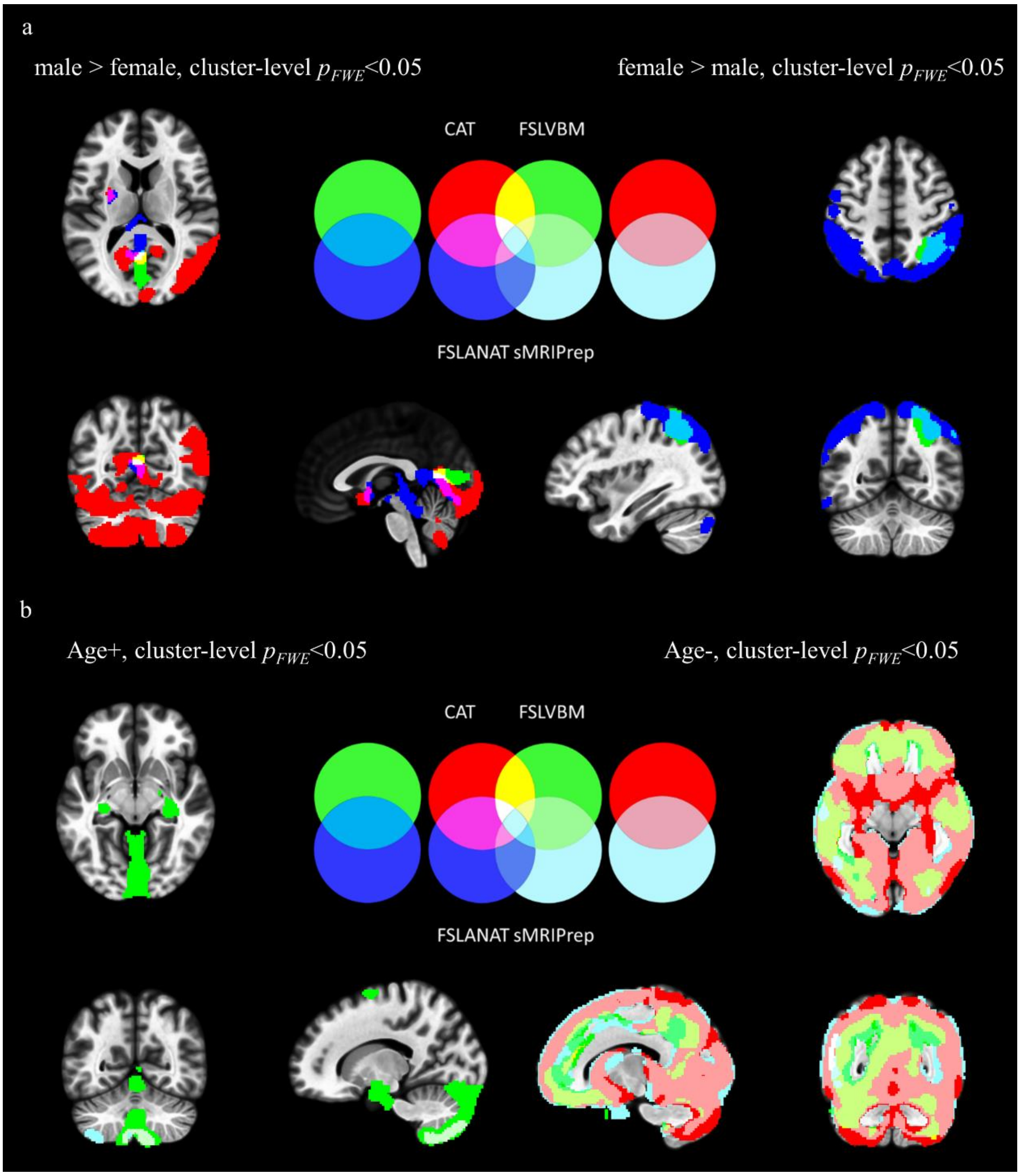

Fig. S5. Results regarding sex differences (a) and age associations (b) after excluding template effect.

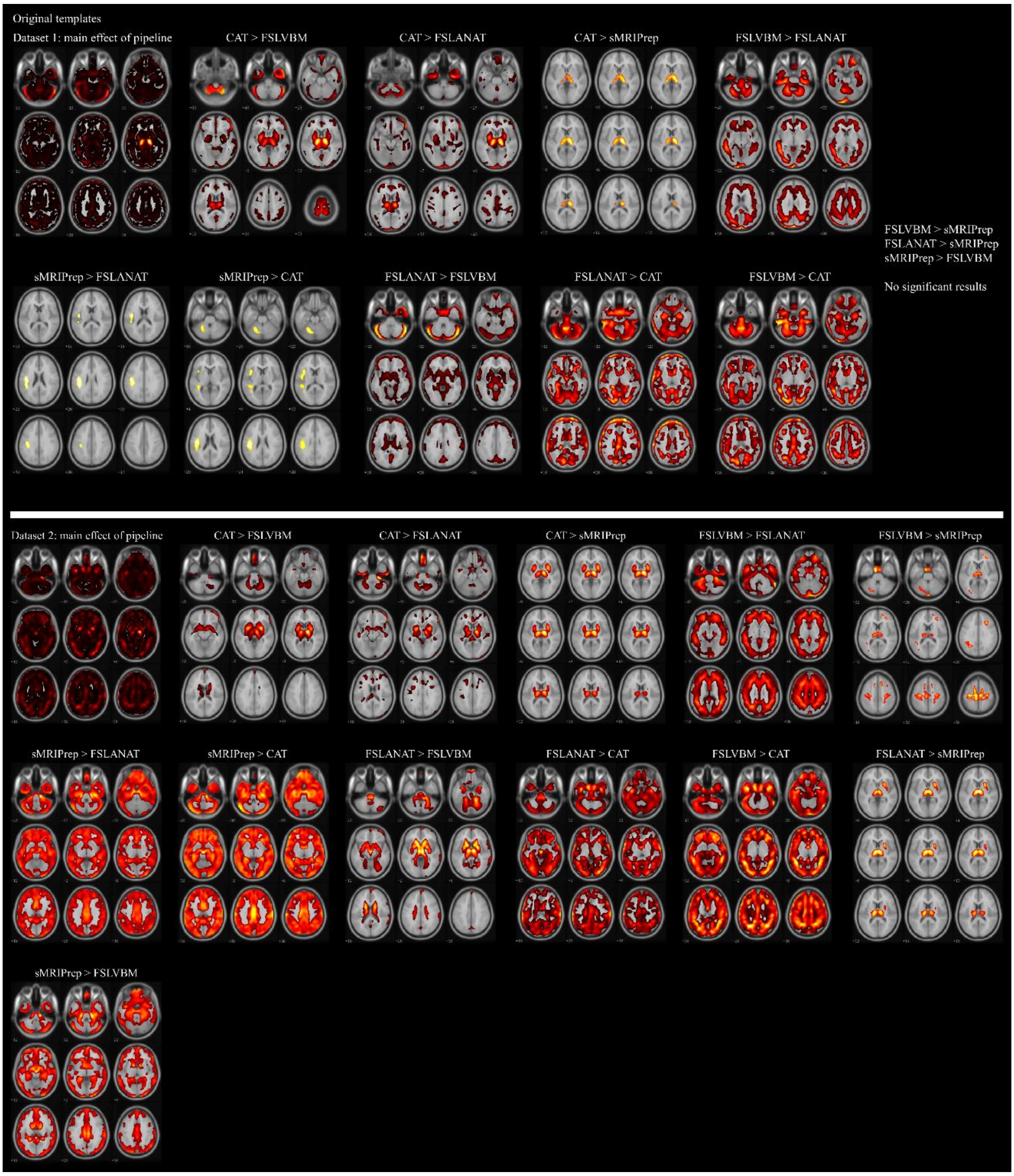

Fig. S6. Direct comparisons between pipelines using the original preprocessed data (both dataset 1 and 2). All results passed cluster level $p_{FWE}<0.05$.

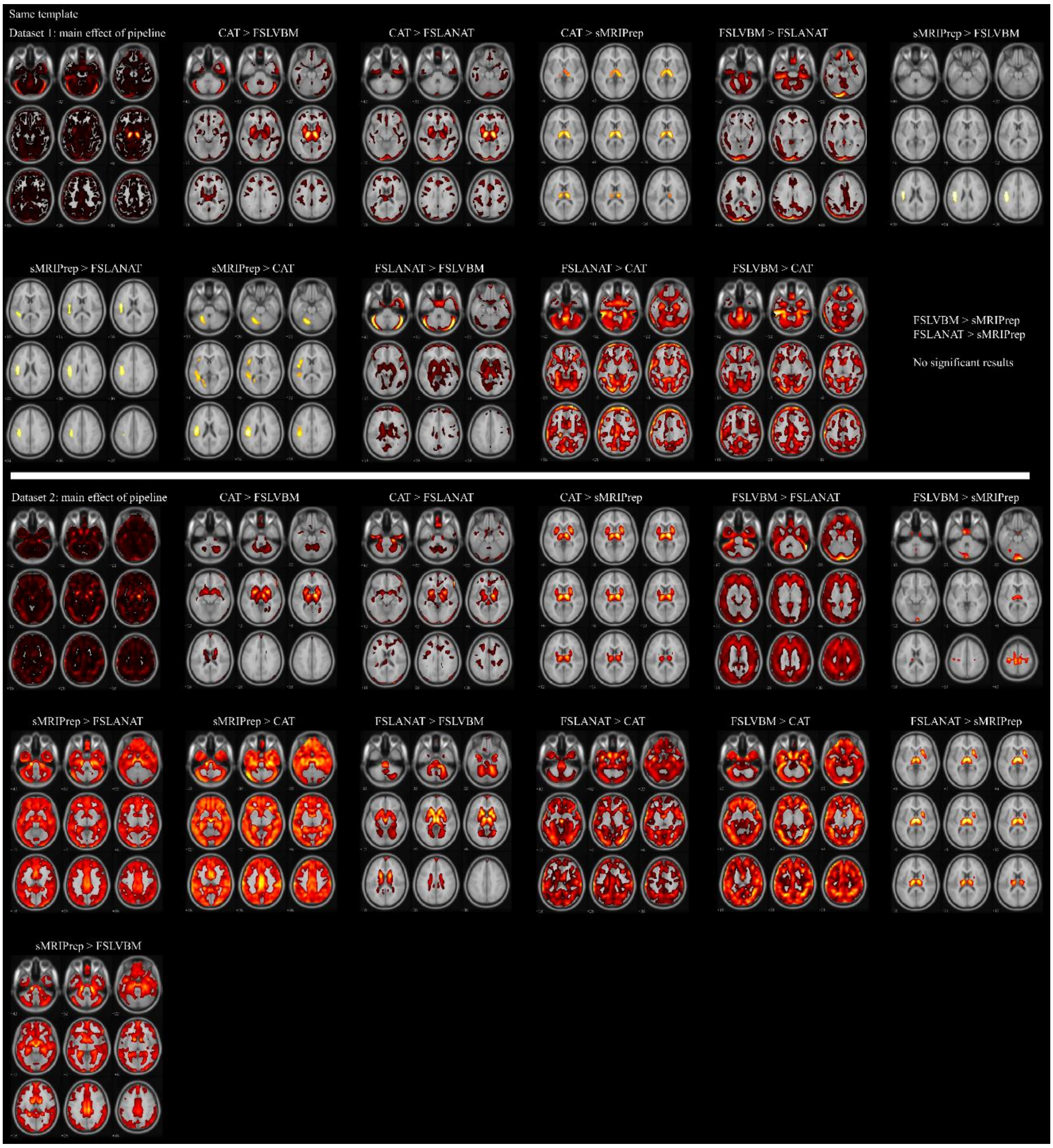

Fig. S7. Direct comparisons between pipelines using the preprocessed data with the same template across pipelines (both dataset 1 and 2). All results passed cluster level $p_{FWE}<0.05$.

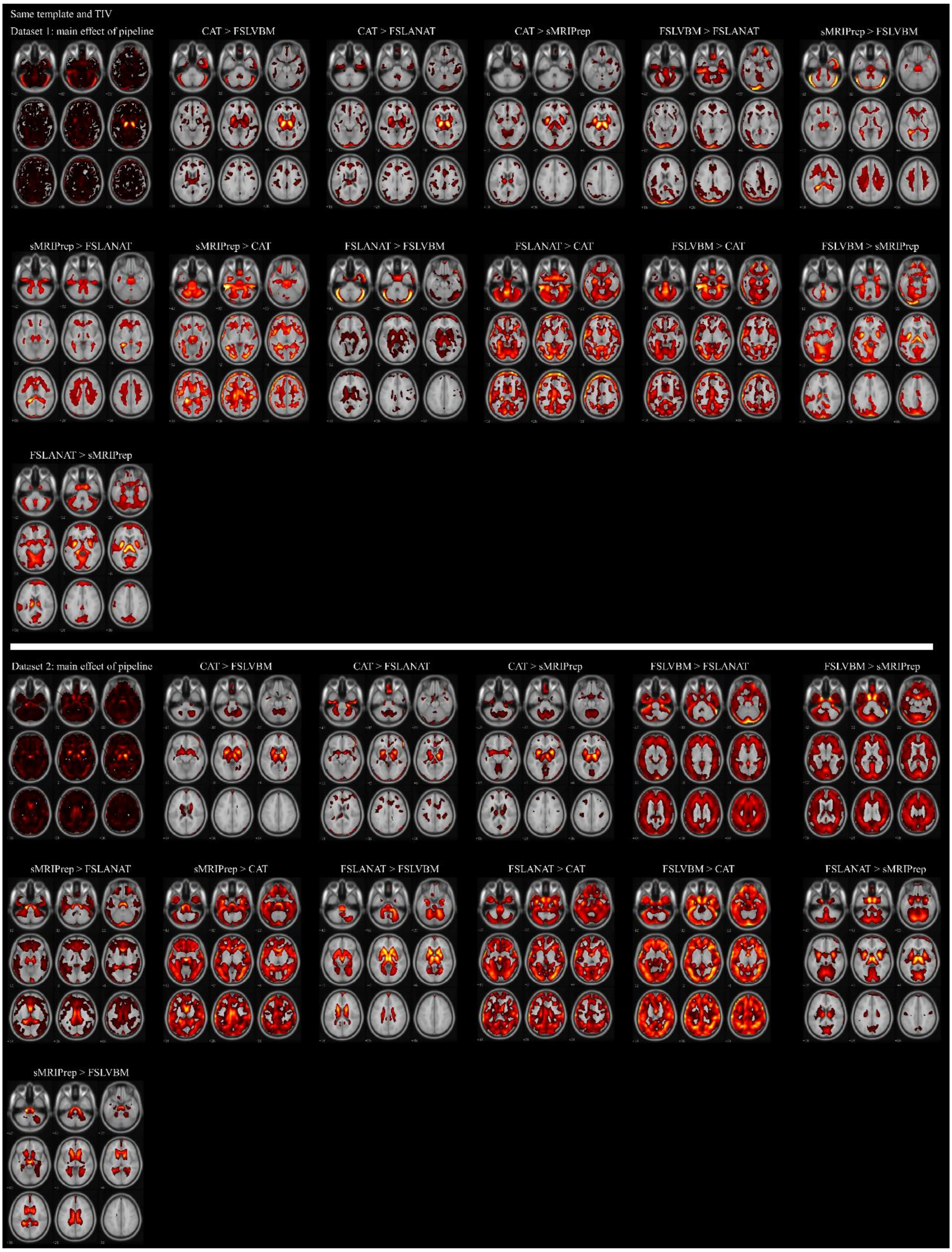

Fig. S8. Direct comparisons between pipelines using the preprocessed data with the same template and the same TIV calculation (both dataset 1 and 2). All results passed cluster level p_{FWE}<0.05.

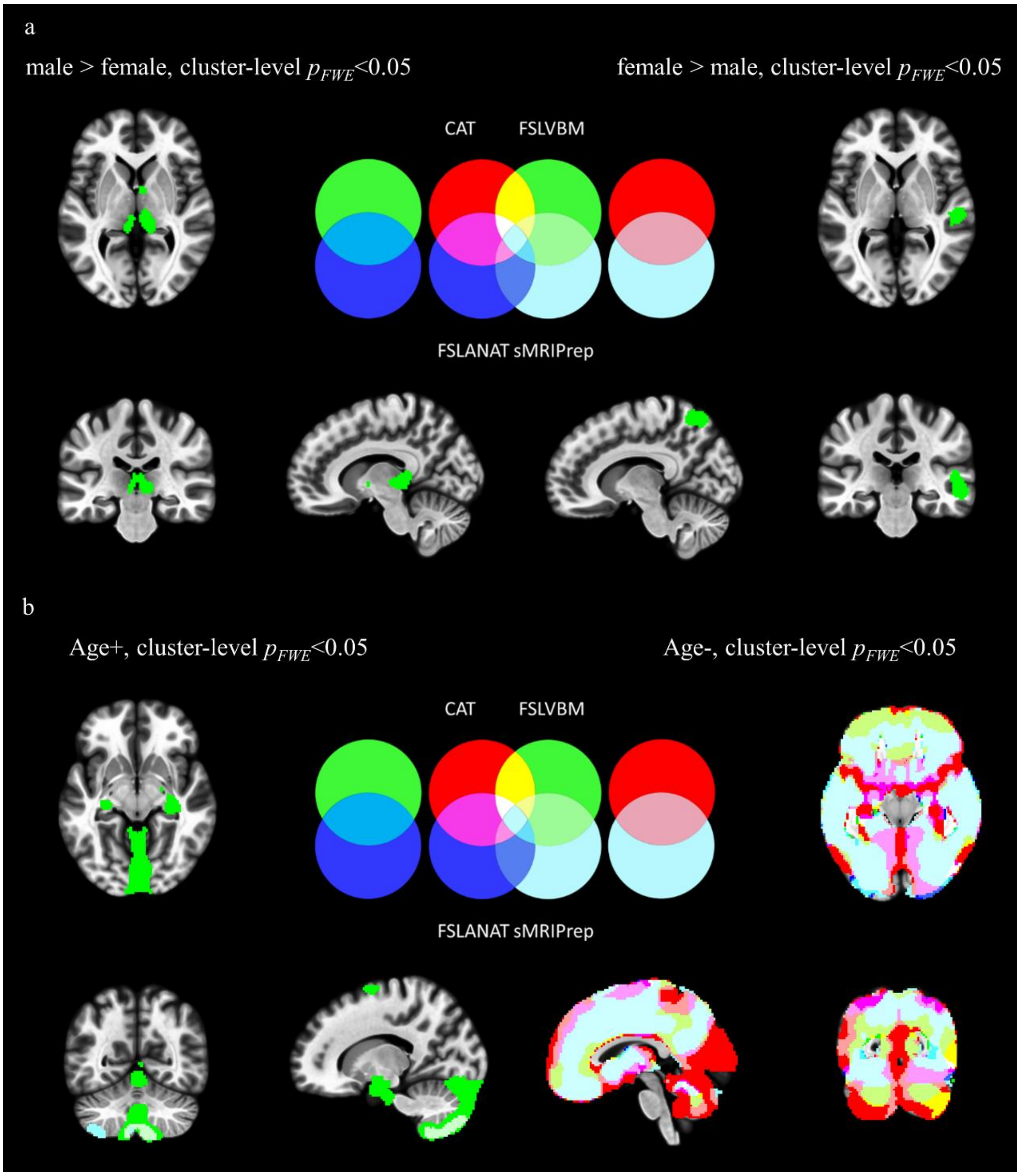

Fig. S9. Results regarding sex differences (a) and age associations (b) after excluding template effect and low spatial similarity data.

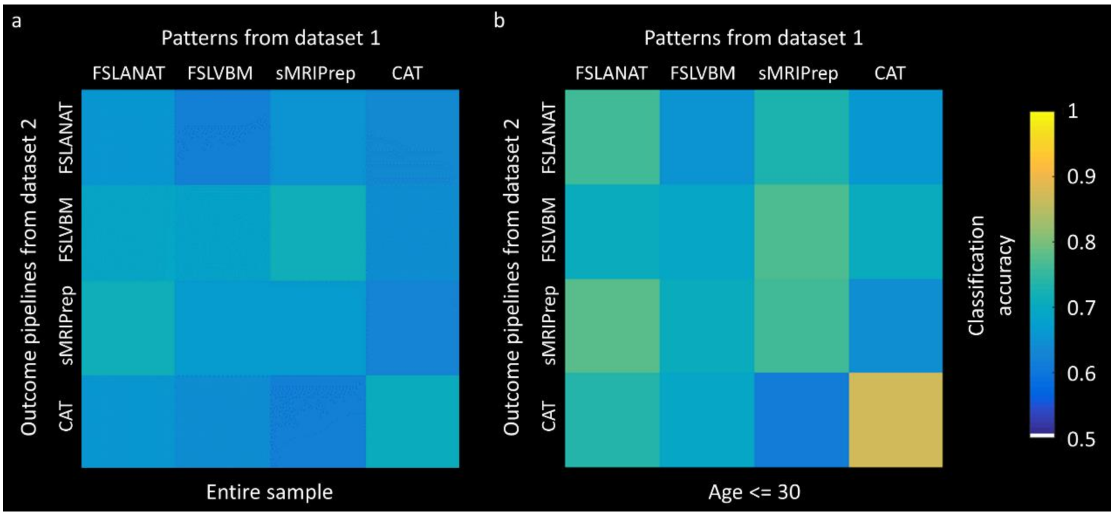

Fig. S10. Classification accuracy of patterns from dataset 1 on dataset 2 for (a) the entire sample, and (b) only age <= 30 (n = 159, female = 99).

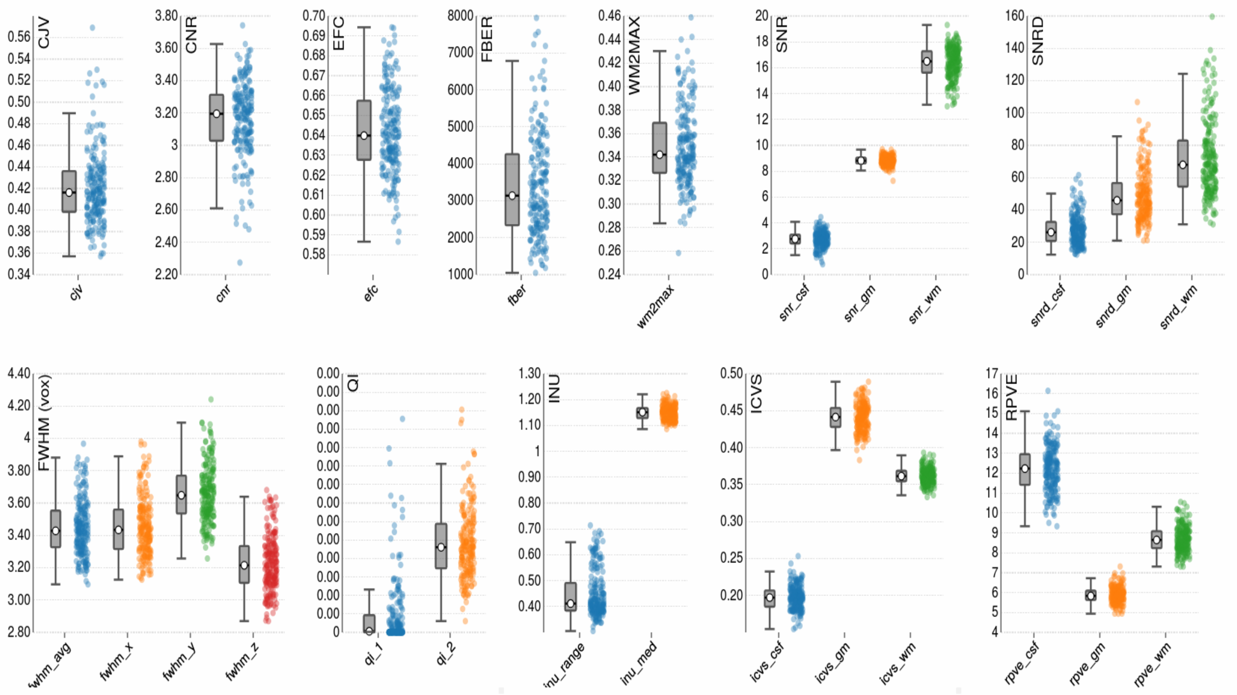

Fig. S11. Quality metrics for dataset 1.

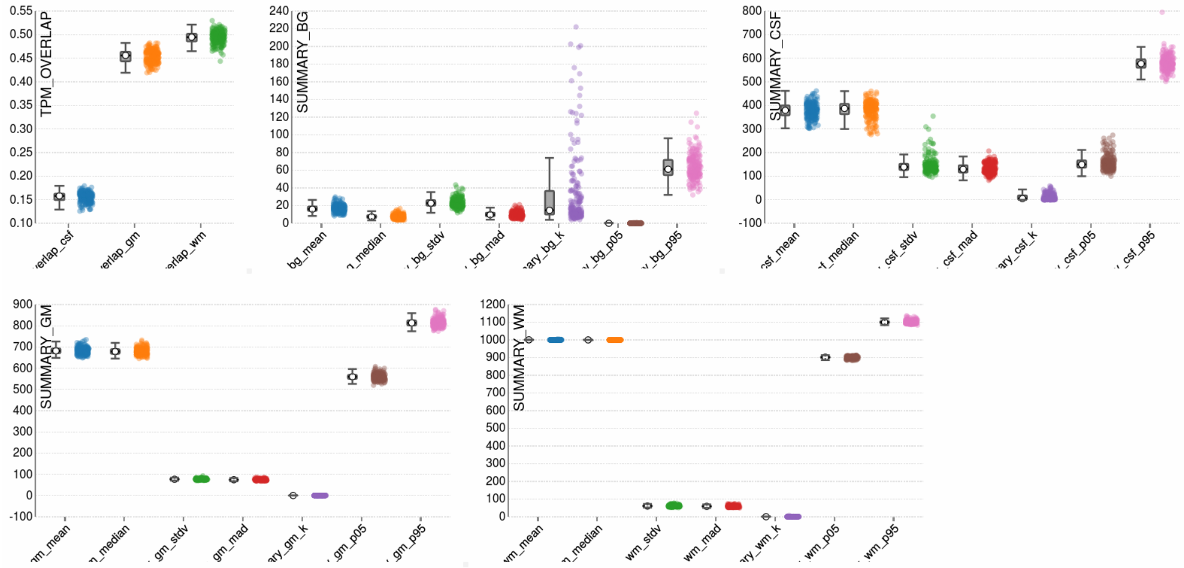

Fig. S12. Quality metrics for dataset 1.

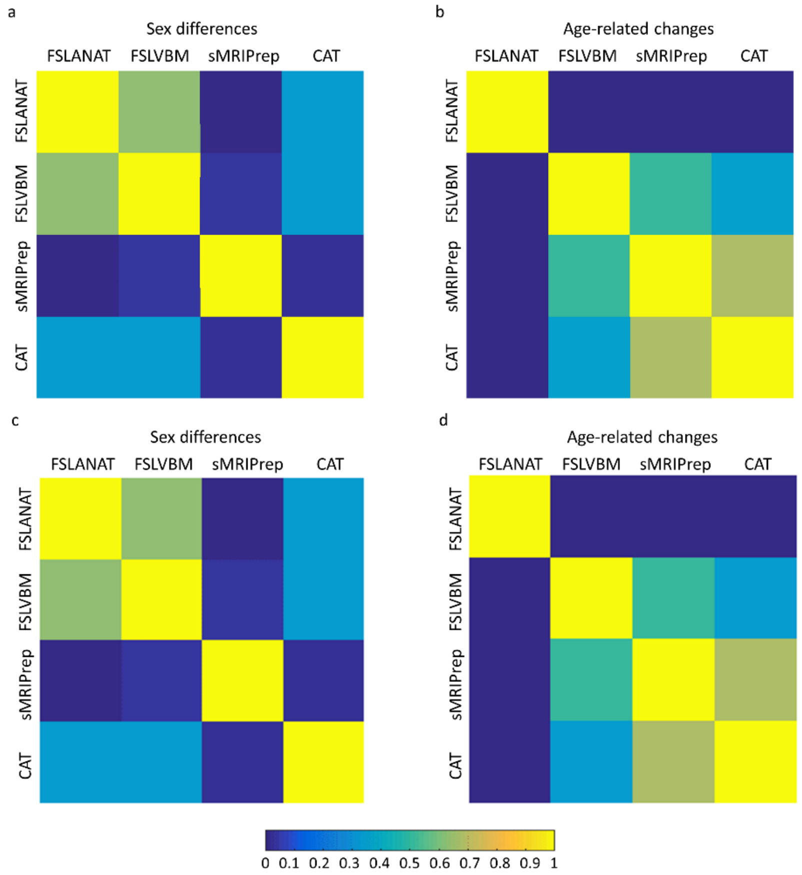

Fig. S13. Variability of unthresholded statistical maps. The correlation values between whole-brain unthresholded statistical maps of four pipelines were computed respectively for (a, c) sex differences, and (b, d) age-related effects. The different MNI templates do not affect the results between a/b using East Asian template, and c/d using Caucasian template. Only positive values are showed for display purpose.

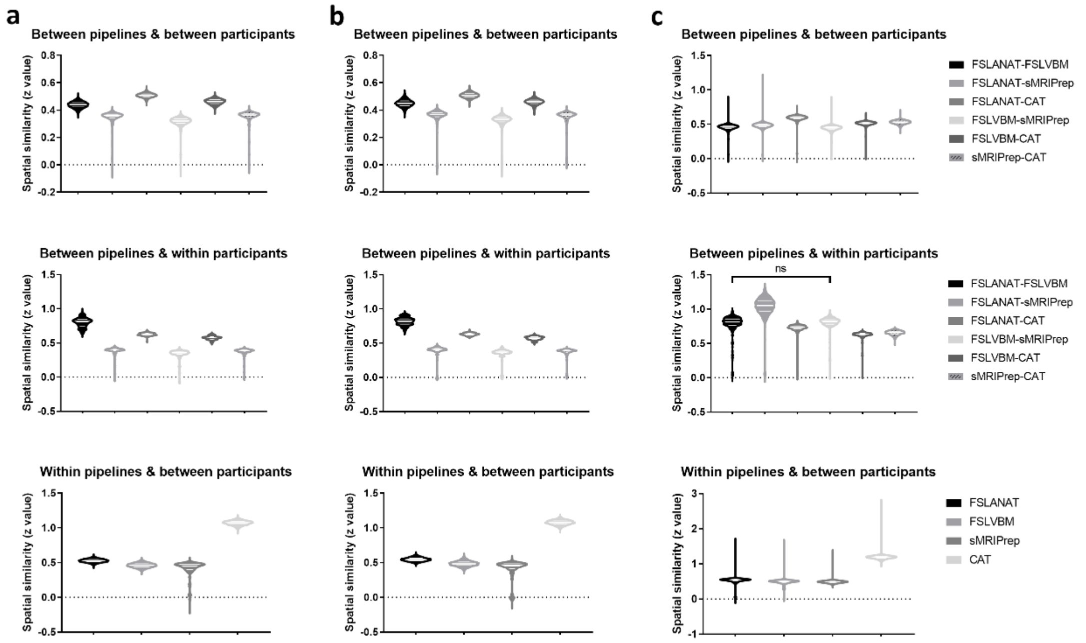

Fig. S14. The mean similarity and SD of different pipelines and pipelines' pairs for the unsmoothed data. The column a represents males, column b represents females, from dataset 1; column c represents dataset 2.

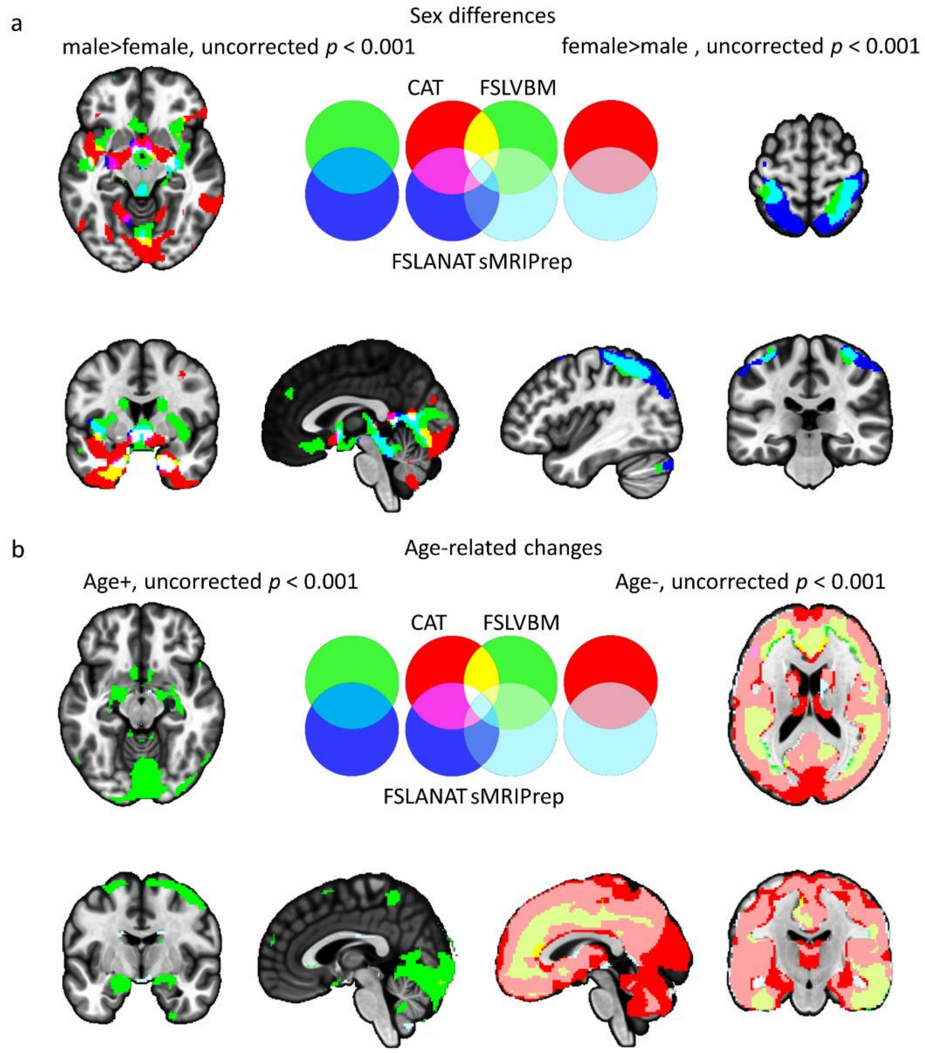

Fig. S15. Similarities and dissimilarities between the pipelines with respect to determining GMV sex differences and age-related GMV changes. Displayed in a and b are results from parametric statistic overlaps at uncorrected $p < 0.001$. The left panels of a display results for the male>female contrast. The right panels of a correspond to the female>male contrast. The left panels of b depicts brain regions with increasing GMV with age. The right panels of b depict decreases with age. Red = CAT, green = FSLVBM, blue = FSLANAT, light blue = sMRIPrep, other colors visualize the overlap between the results.

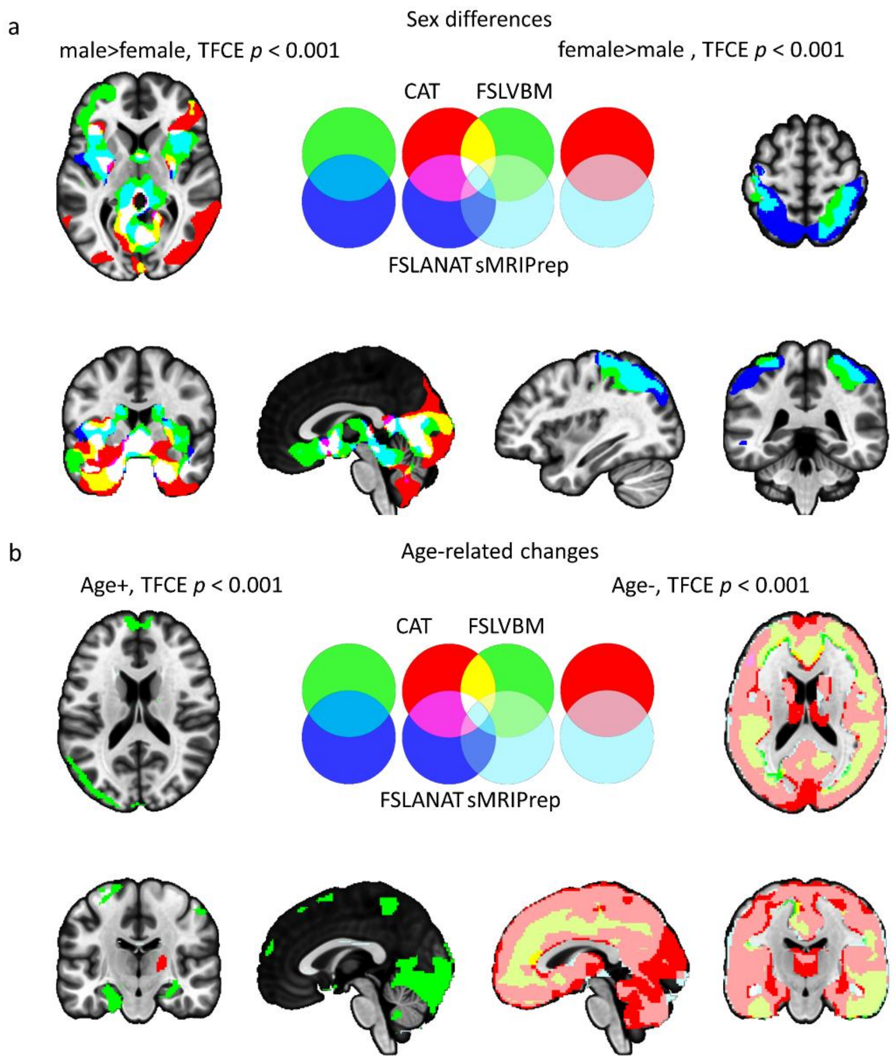

Fig. S16. Similarities and dissimilarities between the pipelines with respect to determining GMV sex differences. Displayed of a and b are results from non-parametric statistics (TFCE with 5,000 permutations) overlapping at $p < 0.001$. The left panels of a display results for the male>female contrast. The right panels of a correspond to the female>male contrast. The left panels of b depicts brain regions with increasing GMV with age. The right panels of b depict decreases with age. Red = CAT, green = FSLVBM, blue = FSLANAT, light blue = sMRIPrep, other colors visualize the overlap between the results.

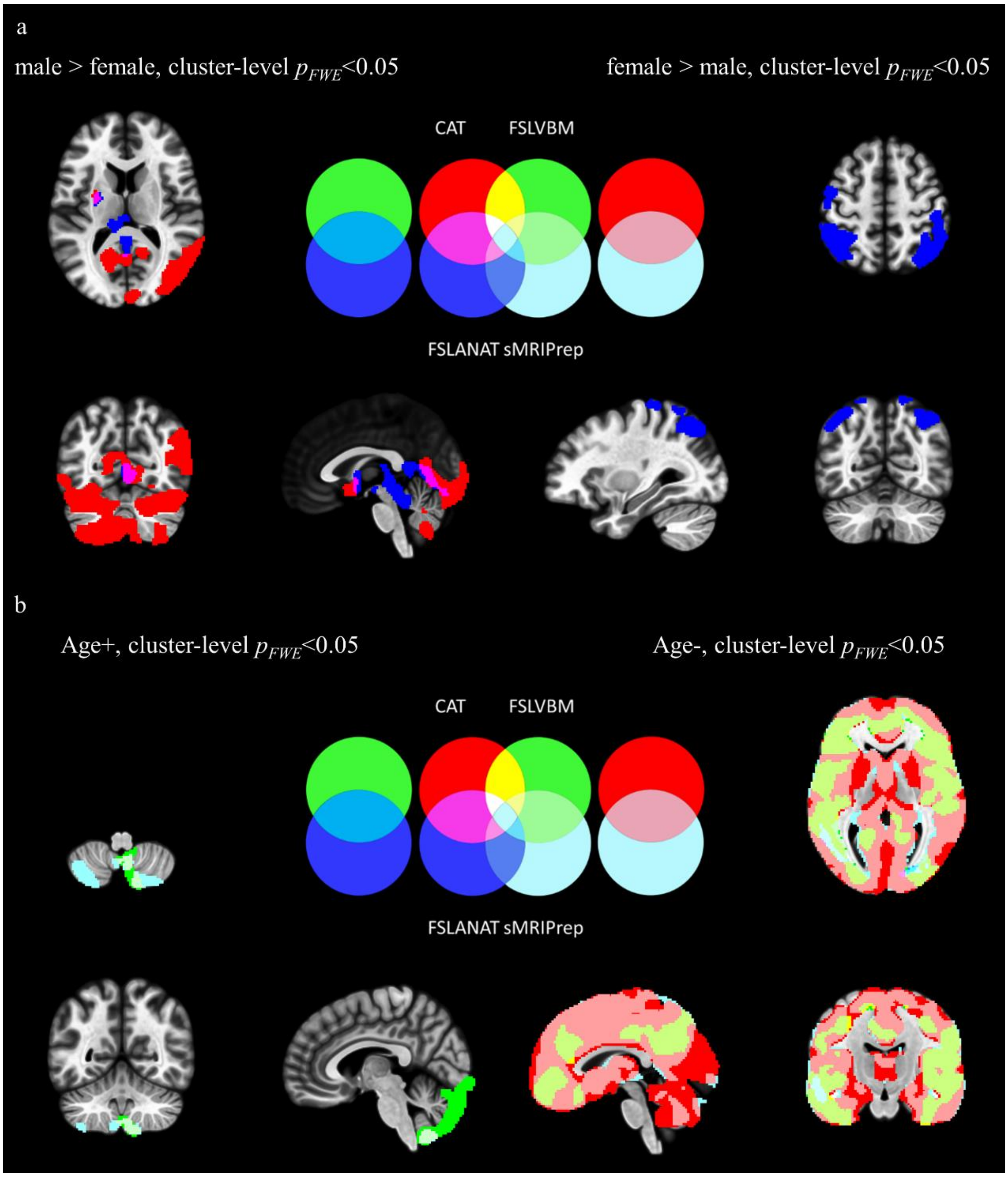

Fig. S17. Results regarding sex differences (a) and age associations (b) after excluding template effect and TIV calculation.

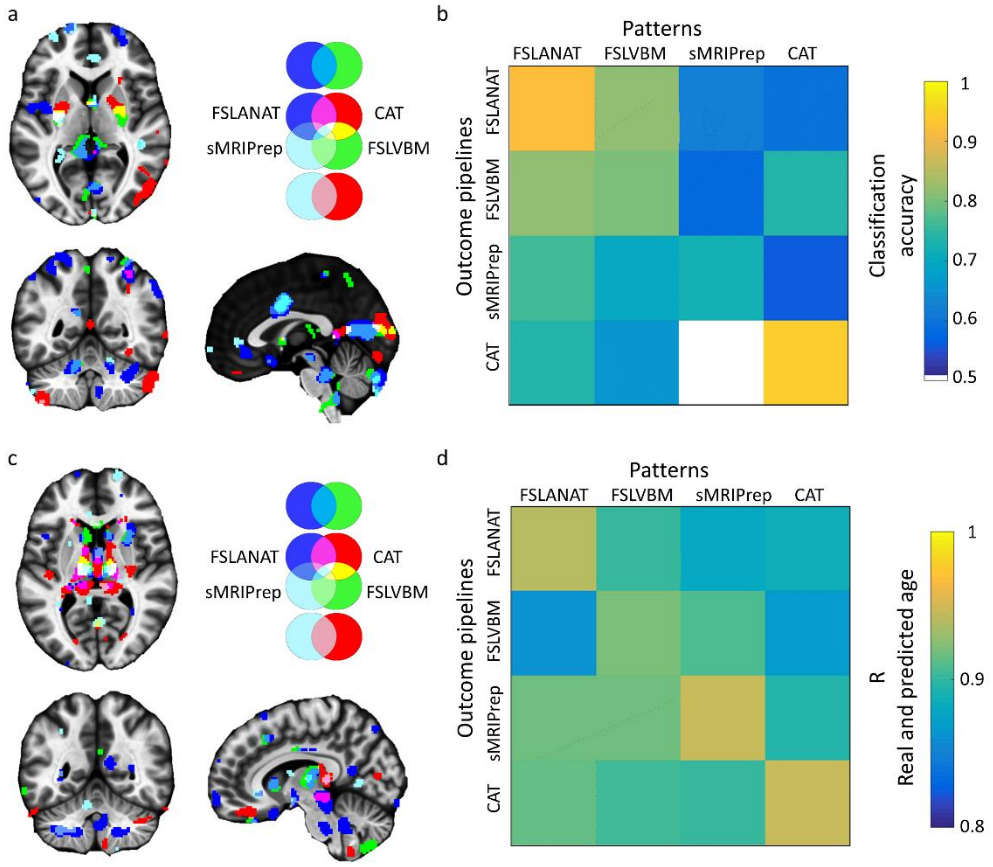

Fig. S18. Distributions and predicted performances of stable prediction patterns for (a, b) sex and (c, d) age after excluding template effect.

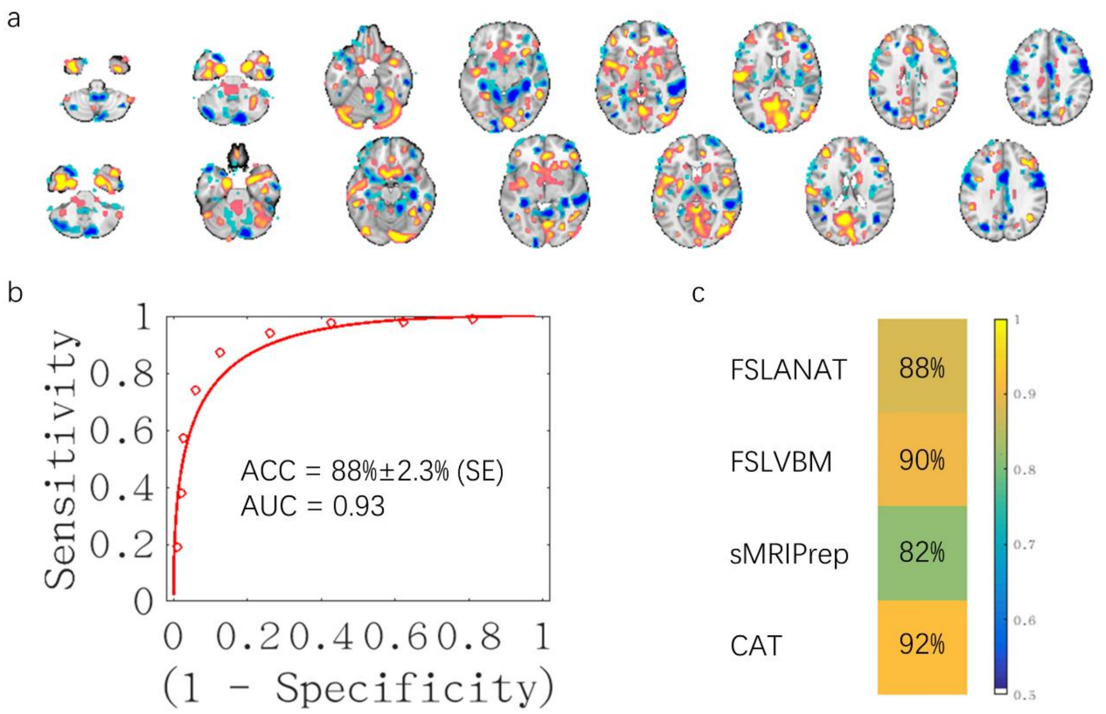

Fig. S19. Distributions and predicted performances of stable prediction patterns for sex.

Supplemental tables

Table S1. Multiple comparisons for between-pipelines and between-participants spatial similarity of male

Bonferroni's multiple comparisons test	Mean Diff.	95.00% CI of diff.	Adjusted P Value
FSLANAT-FSLVBM vs. FSLANAT-sMRIPrep	0.09092	0.08494 to 0.09690	<0.0001
FSLANAT-FSLVBM vs. FSLANAT-CAT	-0.03750	-0.04013 to -0.03487	<0.0001
FSLANAT-FSLVBM vs. FSLVBM-sMRIPrep	0.1520	0.1461 to 0.1578	<0.0001
FSLANAT-FSLVBM vs. FSLVBM-CAT	0.03990	0.03669 to 0.04311	<0.0001
FSLANAT-FSLVBM vs. sMRIPrep-CAT	0.1809	0.1752 to 0.1866	<0.0001
FSLANAT-sMRIPrep vs. FSLANAT-CAT	-0.1284	-0.1339 to -0.1229	<0.0001
FSLANAT-sMRIPrep vs. FSLVBM-sMRIPrep	0.06105	0.05878 to 0.06332	<0.0001
FSLANAT-sMRIPrep vs. FSLVBM-CAT	-0.05102	-0.05684 to -0.04520	<0.0001
FSLANAT-sMRIPrep vs. sMRIPrep-CAT	0.08998	0.08254 to 0.09741	<0.0001
FSLANAT-CAT vs. FSLVBM-sMRIPrep	0.1895	0.1841 to 0.1948	<0.0001
FSLANAT-CAT vs. FSLVBM-CAT	0.07740	0.07531 to 0.07949	<0.0001
FSLANAT-CAT vs. sMRIPrep-CAT	0.2184	0.2133 to 0.2235	<0.0001
FSLVBM-sMRIPrep vs. FSLVBM-CAT	-0.1121	-0.1171 to -0.1070	<0.0001
FSLVBM-sMRIPrep vs. sMRIPrep-CAT	0.02893	0.02166 to 0.03620	<0.0001
FSLVBM-CAT vs. sMRIPrep-CAT	0.1410	0.1356 to 0.1464	<0.0001

Table S2. Multiple comparisons for between-pipelines and between-participants spatial similarity of female

Bonferroni's multiple comparisons test	Mean Diff.	95.00% CI of diff.	Adjusted P Value
FSLANAT-FSLVBM vs. FSLANAT-sMRIPrep	0.1005	0.09391 to 0.1071	<0.0001
FSLANAT-FSLVBM vs. FSLANAT-CAT	0.007626	0.005233 to 0.01002	<0.0001
FSLANAT-FSLVBM vs. FSLVBM-sMRIPrep	0.1463	0.1401 to 0.1525	<0.0001
FSLANAT-FSLVBM vs. FSLVBM-CAT	0.07866	0.07588 to 0.08144	<0.0001
FSLANAT-FSLVBM vs. sMRIPrep-CAT	0.2164	0.2105 to 0.2224	<0.0001
FSLANAT-sMRIPrep vs. FSLANAT-CAT	-0.09289	-0.09950 to -0.08628	<0.0001
FSLANAT-sMRIPrep vs. FSLVBM-sMRIPrep	0.04578	0.04396 to 0.04759	<0.0001
FSLANAT-sMRIPrep vs. FSLVBM-CAT	-0.02185	-0.02864 to -0.01506	<0.0001
FSLANAT-sMRIPrep vs. sMRIPrep-CAT	0.1159	0.1074 to 0.1245	<0.0001
FSLANAT-CAT vs. FSLVBM-sMRIPrep	0.1387	0.1325 to 0.1448	<0.0001
FSLANAT-CAT vs. FSLVBM-CAT	0.07104	0.06956 to 0.07251	<0.0001
FSLANAT-CAT vs. sMRIPrep-CAT	0.2088	0.2034 to 0.2142	<0.0001
FSLVBM-sMRIPrep vs. FSLVBM-CAT	-0.06763	-0.07371 to -0.06155	<0.0001
FSLVBM-sMRIPrep vs. sMRIPrep-CAT	0.07014	0.06203 to 0.07825	<0.0001
FSLVBM-CAT vs. sMRIPrep-CAT	0.1378	0.1322 to 0.1434	<0.0001

Table S3. Multiple comparisons for between-pipelines and within-participants spatial similarity of male

Bonferroni's multiple comparisons test	Mean Diff.	95.00% CI of diff.	Adjusted P Value
FSLANAT-FSLVBM vs. FSLANAT-sMRIPrep	0.3494	0.2624 to 0.4364	<0.0001
FSLANAT-FSLVBM vs. FSLANAT-CAT	0.1633	0.1119 to 0.2146	<0.0001
FSLANAT-FSLVBM vs. FSLVBM-sMRIPrep	0.4362	0.3638 to 0.5087	<0.0001
FSLANAT-FSLVBM vs. FSLVBM-CAT	0.2816	0.2475 to 0.3157	<0.0001
FSLANAT-FSLVBM vs. sMRIPrep-CAT	0.5125	0.4334 to 0.5916	<0.0001
FSLANAT-sMRIPrep vs. FSLANAT-CAT	-0.1861	-0.2567 to -0.1155	<0.0001
FSLANAT-sMRIPrep vs. FSLVBM-sMRIPrep	0.08682	0.05555 to 0.1181	<0.0001
FSLANAT-sMRIPrep vs. FSLVBM-CAT	-0.06780	-0.1431 to 0.007462	0.1190
FSLANAT-sMRIPrep vs. sMRIPrep-CAT	0.1631	0.1453 to 0.1809	<0.0001
FSLANAT-CAT vs. FSLVBM-sMRIPrep	0.2729	0.2039 to 0.3419	<0.0001
FSLANAT-CAT vs. FSLVBM-CAT	0.1183	0.08949 to 0.1471	<0.0001
FSLANAT-CAT vs. sMRIPrep-CAT	0.3492	0.2906 to 0.4077	<0.0001
FSLVBM-sMRIPrep vs. FSLVBM-CAT	-0.1546	-0.2189 to -0.09033	<0.0001
FSLVBM-sMRIPrep vs. sMRIPrep-CAT	0.07626	0.04582 to 0.1067	<0.0001
FSLVBM-CAT vs. sMRIPrep-CAT	0.2309	0.1671 to 0.2946	<0.0001

Table S4. Multiple comparisons for between-pipelines and within-participants spatial similarity of female

Bonferroni's multiple comparisons test	Mean Diff.	95.00% CI of diff.	Adjusted P Value
FSLANAT-FSLVBM vs. FSLANAT-sMRIPrep	0.3709	0.2862 to 0.4557	<0.0001
FSLANAT-FSLVBM vs. FSLANAT-CAT	0.2375	0.2022 to 0.2728	<0.0001
FSLANAT-FSLVBM vs. FSLVBM-sMRIPrep	0.4454	0.3737 to 0.5170	<0.0001
FSLANAT-FSLVBM vs. FSLVBM-CAT	0.3520	0.3282 to 0.3759	<0.0001
FSLANAT-FSLVBM vs. sMRIPrep-CAT	0.5745	0.5072 to 0.6418	<0.0001
FSLANAT-sMRIPrep vs. FSLANAT-CAT	-0.1335	-0.2144 to -0.05250	<0.0001
FSLANAT-sMRIPrep vs. FSLVBM-sMRIPrep	0.07445	0.05105 to 0.09784	<0.0001
FSLANAT-sMRIPrep vs. FSLVBM-CAT	-0.01890	-0.1022 to 0.06441	>0.9999
FSLANAT-sMRIPrep vs. sMRIPrep-CAT	0.2036	0.1796 to 0.2276	<0.0001
FSLANAT-CAT vs. FSLVBM-sMRIPrep	0.2079	0.1324 to 0.2834	<0.0001
FSLANAT-CAT vs. FSLVBM-CAT	0.1146	0.09476 to 0.1344	<0.0001
FSLANAT-CAT vs. sMRIPrep-CAT	0.3370	0.2767 to 0.3974	<0.0001
FSLVBM-sMRIPrep vs. FSLVBM-CAT	-0.09335	-0.1672 to -0.01951	0.0037
FSLVBM-sMRIPrep vs. sMRIPrep-CAT	0.1291	0.1009 to 0.1574	<0.0001
FSLVBM-CAT vs. sMRIPrep-CAT	0.2225	0.1584 to 0.2865	<0.0001

Table S5. Multiple comparisons for within-pipelines and between-participants spatial similarity of male

Bonferroni's multiple comparisons test	Mean Diff.	95.00% CI of diff.	Adjusted P Value
FSLANAT vs. FSLVBM	0.08878	0.08378 to 0.09378	<0.0001
FSLANAT vs. sMRIPrep	0.2117	0.2010 to 0.2223	<0.0001
FSLANAT vs. CAT	-0.4077	-0.4105 to -0.4048	<0.0001
FSLVBM vs. sMRIPrep	0.1229	0.1112 to 0.1345	<0.0001
FSLVBM vs. CAT	-0.4964	-0.5015 to -0.4914	<0.0001
sMRIPrep vs. CAT	-0.6193	-0.6301 to -0.6085	<0.0001

Table S6. Multiple comparisons for within-pipelines and between-participants spatial similarity of female

Bonferroni's multiple comparisons test	Mean Diff.	95.00% CI of diff.	Adjusted P Value
FSLANAT vs. FSLVBM	0.05479	0.05050 to 0.05909	<0.0001
FSLANAT vs. sMRIPrep	0.2404	0.2275 to 0.2532	<0.0001
FSLANAT vs. CAT	-0.3749	-0.3770 to -0.3727	<0.0001
FSLVBM vs. sMRIPrep	0.1856	0.1720 to 0.1991	<0.0001
FSLVBM vs. CAT	-0.4297	-0.4343 to -0.4250	<0.0001
sMRIPrep vs. CAT	-0.6152	-0.6282 to -0.6022	<0.0001

Table S7. Multiple comparisons for between-pipelines and between-participants spatial similarity of dataset 2

Bonferroni's multiple comparisons test	Mean Diff.	95.00% CI of diff.	Adjusted P Value
FSLANAT-FSLVBM vs. FSLANAT-sMRIPrep	-0.03518	-0.03583 to -0.03453	<0.0001
FSLANAT-FSLVBM vs. FSLANAT-CAT	-0.1328	-0.1336 to -0.1320	<0.0001
FSLANAT-FSLVBM vs. FSLVBM-sMRIPrep	-0.03569	-0.03689 to -0.03449	<0.0001
FSLANAT-FSLVBM vs. FSLVBM-CAT	0.02268	0.02154 to 0.02382	<0.0001
FSLANAT-FSLVBM vs. sMRIPrep-CAT	-0.01869	-0.01971 to -0.01767	<0.0001
FSLANAT-sMRIPrep vs. FSLANAT-CAT	-0.09763	-0.09811 to -0.09715	<0.0001
FSLANAT-sMRIPrep vs. FSLVBM-sMRIPrep	-0.0005106	-0.001524 to 0.0005024	>0.9999
FSLANAT-sMRIPrep vs. FSLVBM-CAT	0.05786	0.05688 to 0.05884	<0.0001
FSLANAT-sMRIPrep vs. sMRIPrep-CAT	0.01649	0.01563 to 0.01734	<0.0001
FSLANAT-CAT vs. FSLVBM-sMRIPrep	0.09712	0.09597 to 0.09827	<0.0001
FSLANAT-CAT vs. FSLVBM-CAT	0.1555	0.1544 to 0.1565	<0.0001
FSLANAT-CAT vs. sMRIPrep-CAT	0.1141	0.1132 to 0.1150	<0.0001
FSLVBM-sMRIPrep vs. FSLVBM-CAT	0.05837	0.05797 to 0.05877	<0.0001
FSLVBM-sMRIPrep vs. sMRIPrep-CAT	0.01700	0.01618 to 0.01781	<0.0001
FSLVBM-CAT vs. sMRIPrep-CAT	-0.04137	-0.04194 to -0.04080	<0.0001

Table S8. Multiple comparisons for between-pipelines and within-participants spatial similarity of dataset 2

Bonferroni's multiple comparisons test	Mean Diff.	95.00% CI of diff.	Adjusted P Value
FSLANAT-FSLVBM vs. FSLANAT-sMRIPrep	-0.1898	-0.2134 to -0.1661	<0.0001
FSLANAT-FSLVBM vs. FSLANAT-CAT	0.01048	-0.01726 to 0.03822	>0.9999
FSLANAT-FSLVBM vs. FSLVBM-sMRIPrep	-0.1046	-0.1304 to -0.07874	<0.0001
FSLANAT-FSLVBM vs. FSLVBM-CAT	0.2493	0.2224 to 0.2762	<0.0001
FSLANAT-FSLVBM vs. sMRIPrep-CAT	0.1713	0.1369 to 0.2057	<0.0001
FSLANAT-sMRIPrep vs. FSLANAT-CAT	0.2002	0.1820 to 0.2185	<0.0001
FSLANAT-sMRIPrep vs. FSLVBM-sMRIPrep	0.08520	0.04686 to 0.1235	<0.0001
FSLANAT-sMRIPrep vs. FSLVBM-CAT	0.4391	0.4058 to 0.4724	<0.0001
FSLANAT-sMRIPrep vs. sMRIPrep-CAT	0.3611	0.3304 to 0.3918	<0.0001
FSLANAT-CAT vs. FSLVBM-sMRIPrep	-0.1150	-0.1523 to -0.07781	<0.0001
FSLANAT-CAT vs. FSLVBM-CAT	0.2388	0.2098 to 0.2678	<0.0001
FSLANAT-CAT vs. sMRIPrep-CAT	0.1608	0.1354 to 0.1863	<0.0001
FSLVBM-sMRIPrep vs. FSLVBM-CAT	0.3539	0.3376 to 0.3702	<0.0001
FSLVBM-sMRIPrep vs. sMRIPrep-CAT	0.2759	0.2479 to 0.3038	<0.0001
FSLVBM-CAT vs. sMRIPrep-CAT	-0.07800	-0.09320 to -0.06281	<0.0001

Table S9. Multiple comparisons for within-pipelines and between-participants spatial similarity of dataset 2

Bonferroni's multiple comparisons test	Mean Diff.	95.00% CI of diff.	Adjusted P Value
FSLANAT vs. FSLVBM	-0.05583	-0.05793 to -0.05372	<0.0001
FSLANAT vs. sMRIPrep	-0.003483	-0.005081 to -0.001885	<0.0001
FSLANAT vs. CAT	-0.4597	-0.4612 to -0.4581	<0.0001
FSLVBM vs. sMRIPrep	0.05234	0.05089 to 0.05379	<0.0001
FSLVBM vs. CAT	-0.4038	-0.4053 to -0.4023	<0.0001
sMRIPrep vs. CAT	-0.4562	-0.4567 to -0.4557	<0.0001

Table S10. Image intraclass correlation coefficient (I2C2) across pipelines

	I2C2	95% CI[1]
Dataset 1		
FSLANAT vs. FSLVBM	0.1802	0.1535 to 0.2062
FSLANAT vs. sMRIPrep	0.0489	0.0190 to 0.1067
FSLANAT vs. CAT	0.2983	0.2711 to 0.3247
FSLVBM vs. sMRIPrep	0.0695	0.0337 to 0.1382
FSLVBM vs. CAT	0.3750	0.3445 to 0.4035
sMRIPrep vs. CAT	0.0994	0.0574 to 0.1901
Across all pipelines	0.1613	0.0996 to 0.2579
Dataset 2		
FSLANAT vs. FSLVBM	0.1180	0.0948 to 0.1441
FSLANAT vs. sMRIPrep	0.1205	0.0982 to 0.1424
FSLANAT vs. CAT	0.2437	0.2019 to 0.2860
FSLVBM vs. sMRIPrep	0.1344	0.1118 to 0.1604
FSLVBM vs. CAT	0.3061	0.2617 to 0.3596
sMRIPrep vs. CAT	0.3439	0.3247 to 0.3634
Across all pipelines	0.2656	0.2357 to 0.2960

[1] 95% confidence interval implemented by 5000 bootstrapping

Table S11. Percent overlap of prediction pattern between the pipelines

	Sex[1]	Age[1]
CAT (unique)	21.40%	19.95%
FSLVBM (unique)	13.35%	20.37%
FSLANAT (unique)	35.22%	28.51%
sMRIPrep (unique)	9.77%	4.73%
CAT ∩ FSLVBM	1.40%	1.97%
CAT ∩ FSLANAT	1.97%	4.23%
CAT ∩ sMRIPrep	0.29%	0.74%
FSLVBM ∩ FSLANAT	7.03%	6.68%
FSLVBM ∩ sMRIPrep	1.03%	1.74%
FSLANAT ∩ sMRIPrep	3.58%	3.04%
CAT ∩ FSLVBM ∩ FSLANAT	1.03%	2.14%
CAT ∩ FSLVBM ∩ sMRIPrep	0.37%	0.07%
CAT ∩ FSLANAT ∩ sMRIPrep	0.05%	1.04%
FSLVBM ∩ FSLANAT ∩ sMRIPrep	3.19%	2.39%
CAT ∩ FSLVBM ∩ FSLANAT ∩ sMRIPrep	0.31%	2.39%

[1] Bootstrapping test, p_{FDR}<0.05

Table S12. Prediction performance of sex between the pipelines

ACC (SE) [1], AUC[2]	FSLANAT	FSLVBM	sMRIPrep	CAT
FSLANAT	92% (3.8), 0.98	86% (4.9), 0.92	62% (6.9), 0.74	58% (7.0), 0.60
FSLVBM	90% (4.2), 0.96	88% (4.6), 0.94	50% (7.1), 0.61	74% (6.2), 0.84
sMRIPrep	80% (5.7), 0.89	76% (6.0), 0.86	68% (6.6), 0.77	58% (7.0), 0.62
CAT	72% (6.3), 0.79	76% (6.0), 0.79	14% (4.9), 0.09	94% (3.4), 0.99

[1] accuracy (stand error)

[2] nonparametric area under the curve (AUC)

Table S13. Cohen's d for each classification of male and female

	FSLANAT	FSLVBM	sMRIPrep	CAT
Testing sample from dataset 1				
FSLANAT	2.0260	1.4821	0.5963	0.1392
FSLVBM	1.4589	1.4798	0.2930	0.6472
SMRIPrep	0.6437	0.7231	0.2967	0.1693
CAT	0.7810	0.7402	-1.4909	2.2815
Testing sample from dataset 2 (age<=30)				
FSLANAT	1.0091	0.5780	0.4651	0.4242
FSLVBM	0.8998	0.8162	0.6787	0.2237
SMRIPrep	1.2160	0.8602	1.1182	0.2635
CAT	0.7471	0.7350	-1.0130	2.2062

Table S14. Percent overlap of age associated negative GMV-changes between the pipelines controlling for template effect and sample homogeneity

	Negative association Parametric[1]
CAT (unique)	11.84%
FSLVBM (unique)	1.12%
FSLANAT (unique)	0.53%
sMRIPrep (unique)	1.28%
CAT ∩ FSLVBM	1.87%
CAT ∩ FSLANAT	3.17%
CAT ∩ sMRIPrep	6.46%
FSLVBM ∩ FSLANAT	0.19%
FSLVBM ∩ sMRIPrep	0.81%
FSLANAT ∩ sMRIPrep	0.57%
CAT ∩ FSLVBM ∩ FSLANAT	0.72
CAT ∩ FSLVBM ∩ sMRIPrep	12.18%
CAT ∩ FSLANAT ∩ sMRIPrep	9.09%
FSLVBM ∩ FSLANAT ∩ sMRIPrep	1.57
CAT ∩ FSLVBM ∩ FSLANAT ∩ sMRIPrep	48.60%

[1]cluster-level p_{FWE}<0.05

Table S15. Prediction performance of sex between the pipelines (independent test dataset) with common template

ACC (SE) [1], AUC[2]	FSLANAT	FSLVBM	sMRIPrep	CAT
FSLANAT	92% (3.8), 0.97	82% (5.4), 0.87	62% (6.9), 0.76	60% (6.9), 0.59
FSLVBM	82% (5.4), 0.93	80% (5.7), 0.88	58% (7.0), 0.65	74% (6.2), 0.84
sMRIPrep	76% (6.0), 0.85	70% (6.5), 0.73	72% (6.3), 0.77	56% (7.0), 0.58
CAT	74% (6.2), 0.77	66% (6.7), 0.69	14% (4.9), 0.08	94% (3.4), 0.99

[1] accuracy (stand error)

[2] nonparametric area under the curve (AUC)

Table S16. Percent overlap of prediction pattern between the pipelines with common template

	Sex	Age
CAT (unique)	22.49%	24.38%
FSLVBM (unique)	11.20%	9.47%
FSLANAT (unique)	35.30%	31.96%
sMRIPrep (unique)	9.09%	6.70%
CAT ∩ FSLVBM	1.28%	1.11%
CAT ∩ FSLANAT	1.67%	5.13%
CAT ∩ sMRIPrep	0.17%	0.66%
FSLVBM ∩ FSLANAT	10.93%	5.59%
FSLVBM ∩ sMRIPrep	0.27%	2.10%
FSLANAT ∩ sMRIPrep	2.16%	4.33%
CAT ∩ FSLVBM ∩ FSLANAT	1.69%	2.23%
CAT ∩ FSLVBM ∩ sMRIPrep	0.11%	0.23%
CAT ∩ FSLANAT ∩ sMRIPrep	0.02%	1.22%
FSLVBM ∩ FSLANAT ∩ sMRIPrep	3.44%	2.16%
CAT ∩ FSLVBM ∩ FSLANAT ∩ sMRIPrep	0.16%	2.73%

[1] Bootstrapping test, $p_{FDR}<0.05$

Supplementary References

1 Wei, D. *et al.* Structural and functional brain scans from the cross-sectional Southwest University adult lifespan dataset. *Sci Data* **5**, 180134, doi:10.1038/sdata.2018.134 (2018).

2 Yan, C. G., Wang, X. D., Zuo, X. N. & Zang, Y. F. DPABI: Data Processing & Analysis for (Resting-State) Brain Imaging. *Neuroinformatics* **14**, 339-351, doi:10.1007/s12021-016-9299-4 (2016).

3 Ashburner, J. & Friston, K. J. Diffeomorphic registration using geodesic shooting and Gauss-Newton optimisation. *Neuroimage* **55**, 954-967, doi:10.1016/j.neuroimage.2010.12.049 (2011).
