# Supplementary material for: Choice of Voxel-based Morphometry processing pipeline drives variability in the location of neuroanatomical brain markers
Source: Commun Biol. 2022 Sep 6;5:913. doi: 10.1038/s42003-022-03880-1 (PMC9448776; doi:10.1038/s42003-022-03880-1)
Supplement: Supplementary file 2 — Reporting Summary [file 42003_2022_3880_MOESM2_ESM.pdf]

nature portfolio

Corresponding author(s): Benjamin Becker & Xinqi Zhou

Last updated by author(s): Jul 27, 2022

Reporting Summary

Nature Portfolio wishes to improve the reproducibility of the work that we publish. This form provides structure for consistency and transparency in reporting. For further information on Nature Portfolio policies, see our Editorial Policies and the Editorial Policy Checklist.

Statistics

For all statistical analyses, confirm that the following items are present in the figure legend, table legend, main text, or Methods section.

n/a	Confirmed	
☐	☒	The exact sample size (*n*) for each experimental group/condition, given as a discrete number and unit of measurement
☐	☒	A statement on whether measurements were taken from distinct samples or whether the same sample was measured repeatedly
☐	☒	The statistical test(s) used AND whether they are one- or two-sided<br>*Only common tests should be described solely by name; describe more complex techniques in the Methods section.*
☐	☒	A description of all covariates tested
☐	☒	A description of any assumptions or corrections, such as tests of normality and adjustment for multiple comparisons
☐	☒	A full description of the statistical parameters including central tendency (e.g. means) or other basic estimates (e.g. regression coefficient) AND variation (e.g. standard deviation) or associated estimates of uncertainty (e.g. confidence intervals)
☐	☒	For null hypothesis testing, the test statistic (e.g. *F*, *t*, *r*) with confidence intervals, effect sizes, degrees of freedom and *P* value noted<br>*Give P values as exact values whenever suitable.*
☒	☐	For Bayesian analysis, information on the choice of priors and Markov chain Monte Carlo settings
☒	☐	For hierarchical and complex designs, identification of the appropriate level for tests and full reporting of outcomes
☒	☐	Estimates of effect sizes (e.g. Cohen's *d*, Pearson's *r*), indicating how they were calculated

Our web collection on statistics for biologists contains articles on many of the points above.

Software and code

Policy information about availability of computer code

Data collection	No software was used.
Data analysis	The following list includes all softwares used in the current study: (1) CAT12.7 r1720, (2) SPM12 v7219, (3) TFCE 156, (4)FSL v6.0, (5) sMRIPrep 0.6.2, (6) MRIQC v0.15.2, (7) MATLAB R2016b, (8) BrainNet Viewer 1.63, (9) CANLab Core Tools (https://github.com/canlab), (10) I2C2 (http://www.biostat.jhsph.edu/~ccrainic/software.html), (11) code to calculate TIV (http://enigma.ini.usc.edu/protocols/imaging-protocols/protocol-for-brain-and-intracranial-volumes/#fsl), (12) DPABI v4.2, (13) Mango 4.1 (1531), and (14) GraphPad Prism 6

For manuscripts utilizing custom algorithms or software that are central to the research but not yet described in published literature, software must be made available to editors and reviewers. We strongly encourage code deposition in a community repository (e.g. GitHub). See the Nature Portfolio guidelines for submitting code & software for further information.

Data

Policy information about availability of data

All manuscripts must include a data availability statement. This statement should provide the following information, where applicable:

- Accession codes, unique identifiers, or web links for publicly available datasets
- A description of any restrictions on data availability
- For clinical datasets or third party data, please ensure that the statement adheres to our policy

Unthreshold statistical maps and pattern weight images are available on OSF (https://osf.io/p5b6f/). Other data can be obtained from the corresponding authors upon reasonable request.

Human research participants

Policy information about studies involving human research participants and Sex and Gender in Research.

Reporting on sex and gender	Both male and female were included in the current study and sex was considered as an independent variable in the analysis of dataset 1.
Population characteristics	Dataset 1 included T1-weigthed anatomical data from 200 healthy Chinese participants aged 18-26 years old (mean = 21.45 years old, SD = 2.18; 100 females and 100 males matched for age, details see Liu et al., 2020). Dataset 2 included 494 healthy Chinese participants aged 19-80 years (mean = 45.18 years, SD = 17.44, 187 males) from an openly available dataset (SALD) encompassing T1-weigthed anatomical and resting-state functional MRI data (details please see Wei et al. (2018)).
Recruitment	Dataset 1 is from previous study (Liu et al., 2020) . Dataset 2 is an openly available dataset (Wei et al., 2018).
Ethics oversight	Dataset 1: University of Electronic Science and Technology of China<br>Dataset 2: Southwest University

Note that full information on the approval of the study protocol must also be provided in the manuscript.

Field-specific reporting

Please select the one below that is the best fit for your research. If you are not sure, read the appropriate sections before making your selection.

☒ Life sciences ☐ Behavioural & social sciences ☐ Ecological, evolutionary & environmental sciences

For a reference copy of the document with all sections, see nature.com/documents/nr-reporting-summary-flat.pdf

Life sciences study design

All studies must disclose on these points even when the disclosure is negative.

Sample size	Dataset 1 included T1-weigthed anatomical data from 200 healthy Chinese participants aged 18-26 years old (mean = 21.45 years old, SD = 2.18; 100 females and 100 males matched for age, details see Liu et al., 2020). Dataset 2 included 494 healthy Chinese participants aged 19-80 years (mean = 45.18 years, SD = 17.44, 187 males) from an openly available dataset (SALD) encompassing T1-weigthed anatomical and resting-state functional MRI data (details please see Wei et al. (2018)).
Data exclusions	No data was excluded.
Replication	Comparisons of multi preprocessing pipelines and analysis methods.
Randomization	Not related to the current study. The data collection was done by other studies.
Blinding	Not related to the current study. The data collection was done by other studies.

Reporting for specific materials, systems and methods

We require information from authors about some types of materials, experimental systems and methods used in many studies. Here, indicate whether each material, system or method listed is relevant to your study. If you are not sure if a list item applies to your research, read the appropriate section before selecting a response.

Materials & experimental systems

n/a	Involved in the study
☒	☐ Antibodies
☒	☐ Eukaryotic cell lines
☒	☐ Palaeontology and archaeology
☒	☐ Animals and other organisms
☒	☐ Clinical data
☒	☐ Dual use research of concern

Methods

n/a	Involved in the study
☒	☐ ChIP-seq
☒	☐ Flow cytometry
☐	☒ MRI-based neuroimaging

Magnetic resonance imaging

Experimental design

Design type: T1-weigthed anatomical data acquistion

Design specifications: No functional data

Behavioral performance measures: No behavioral performance measures

Acquisition

Imaging type(s): structural

Field strength: 3T

Sequence & imaging parameters: Both datasets were acquired using validated T1-weighted brain structural acquisition protocols. Dataset 1 was acquired on a 3.0 Tesla GE MR750 system (General Electric Medical Systems, Milwaukee, WI, USA). T1-weighted high-resolution anatomical images were acquired with a spoiled gradient echo pulse sequence, repetition time (TR) = 5.9 ms, echo time (TE) = 2 ms, flip angle = 9°, field of view (FOV) = 256 × 256 mm, acquisition matrix = 256 × 256, thickness = 1 mm, number of slices = 156, voxel size=1×1×1 mm. Dataset 2 was collected using a 3.0-T Siemens Trio MRI scanner (Siemens Medical, Erlangen, Germany). A magnetization-prepared rapid gradient echo (MPRAGE) sequence was used to acquire high-resolution T1-weighted anatomical images (repetition time = 1,900 ms, echo time = 2.52 ms, inversion time = 900 ms, flip angle = 90°, resolution matrix = 256 × 256, slices = 176, thickness = 1.0 mm, and voxel size = 1×1×1 mm) (Wei et al., 2018).

Area of acquisition: whole brain

Diffusion MRI: ☐ Used ☒ Not used

Preprocessing

Preprocessing software: The CAT pipeline was implemented in CAT12.7 running on SPM12 v7219 (Welcome Department of Cognitive Neurology, London, UK, https://www.fil.ion.ucl.ac.uk/spm/software/spm12/). Standard VBM preprocessing protocols of CAT12 as outlined in the CAT12.7 manual were employed in the pipeline. Briefly, the T1-weighted images were bias-corrected, segmented into gray matter (GM), white matter (WM), and cerebrospinal fluid (CSF) using SPM's unified segmentation function for segmentation and initial registration, with additional optimization of the segmentation (e.g. using local adaptive segmentation and adaptive maximum a posterior segmentation) and spatially normalized to the standard Montreal Neurological Institute (MNI) space using the ICBM152 template (East Asian, additional results obtained with the Caucasian template did not affect the results, see supplements Fig. S13) with a voxel size of 2×2×2 mm. GM images were smoothed with three Gaussian kernels with commonly used smoothing kernels (8 mm, 10 mm, and 12mm) at full-width at half maximum (FWHM) for subsequent statistical analysis and total intracranial volume (TIV) was estimated to correct for individual differences in brain size. Default parameters were applied unless indicated otherwise.

Two different default preprocessing pipelines were established in FSL (Jenkinson et al., 2012; Smith et al., 2004): (1) FSLVBM (https://fsl.fmrib.ox.ac.uk/fsl/fslwiki/FSLVBM), and (2) FSLANAT (https://fsl.fmrib.ox.ac.uk/fsl/fslwiki/fsl_anat). The FSLVBM d efault pipeline included the following four steps: First, non-brain tissue was removed using BET (fslvbm_1_bet). Second (fslvbm_2_template), tissue-type segmentation was conducted via the Automated Segmentation Tool (FAST), to segment the images into GM, WM, and CSF. Third, the outcomes were non-linearly registered to the GM ICBM-152 template using the registration tool FNIRT, then creating a study-specific template. Finally, the GM images were non-linearly registered to the study-specific template using FNIRT (fslvbm_3_proc). In contrast, FSLANAT is a general pipeline for processing anatomical images encompassing the following steps (fsl_anat). Of note the processing order is different from FSLVBM and the final outcomes are segmented data in native space. First, all T1-weighted images were reorientated to the standard MNI orientation (fslreorient2std) and automatically cropped (robustfov). Second, bias-field correction for RF/B1-inhomogeneity-correction (FAST) was done. Third, the pipeline did brain-extraction (BET) and tissue-type segmentation (FAST). The calculation of TIV for both FSLANAT and FSLVBM adhered to the protocols provided by the ENIGMA project (http://enigma.ini.usc.edu/protocols/imaging-protocols/protocol-for-brain-and-intracranial-volumes/#fsl).

sMRIPrep 0.6.2 (Esteban et al. (2019), RRID:SCR_016216, https://www.nipreps.org/smriprep/) is a structural MRI data

preprocessing pipeline designed to provide an easily accessible, state-of-the-art interface that is robust to variations in scan acquisition protocols and that requires minimal user input, while providing easily interpretable and comprehensive error and output reporting. The workflow is based on Nipype 1.5.0 (Gorgolewski et al. (2011), RRID:SCR_002502). The similar workflow is also used in fMRIPrep anatomical preprocessing workflow (Esteban et al. (2019), https://fmriprep.org/). In the present study, the T1-weighted (T1w) image was corrected for intensity non-uniformity (INU) with N4BiasFieldCorrection (Tustison et al., 2010), distributed with ANTs 2.2.0 (Avants et al. (2008), RRID:SCR_004757), and used as T1w-reference throughout the workflow. The T1w-reference was then skull-stripped with a Nipype implementation of the antsBrainExtraction.sh workflow (from ANTs), using OASIS30ANTs as target template. Brain tissue segmentation of CSF, WM and GM was performed on the brain-extracted T1w using fast (FSL 5.0.9, RRID:SCR_002823, Zhang et al. (2001)). Considering no TIV estimation is provided by sMRIPrep, the corresponding brain size for the analysis was computed by summarizing the tissue types (GM+WM+CSF).
To reduce further variability induced by spatial normalization the FSLANAT, FSLVBM and sMRIPrep pipelines used the same normalization. In detail, sMRIPrep and FSLANAT were mainly used for segmenting GM, WM, and CSF data, and the data next was integrated into the FSLVBM pipeline. For processing in these pipelines we thus excluded the initial brain-extraction (fslvbm_1_bet) and segmentation (first part of fslvbm_2_template) stages and subjected the segmented GM outcomes (in native space) to fslvbm_2_template and fslvbm_3_proc to produce modulated GM data.
To keep preprocessing consistent within each platform the fslmaths function was used to smooth FSL processed data (FSLVBM, FSLANAT and sMRIPrep) with comparable smoothing kernels (sigma = 3.5, 4.3, 5.2, approximately corresponding to FWHM - 3.5×2.3=8.05≈8, 4.3×2.3=9.89≈10, and 5.2×2.3=11.96≈12) as the CAT data. For the CAT preprocessing, SPM smoothing was conducted with FWHM = 8, 10, and 12 respectively.

Normalization: See above

Normalization template: See above

Noise and artifact removal: See above

Volume censoring: Not applicable

Statistical modeling & inference

Model type and settings: mass univariate (between-groups comparisons, linear regression analysis) and multivariate (Multivariate Pattern Analyses based prediction)

Effect(s) tested: sex differences in gray matter volume, and age-related changes in gray matter volume.

Specify type of analysis: ☒ Whole brain ☐ ROI-based ☐ Both

Statistic type for inference (See Eklund et al. 2016): To account for potential interactions between preprocessing and inferential statistical procedures all univariate analyses in the current study were conducted in SPM12 and across different multiple comparisons corrections. The analyses including conventional statistical parameter tests (threshold at voxel-level $p < 0.001$, and cluster-level pFWE < 0.05 with initial cluster forming voxel-level $p < 0.001$ respectively) as well as threshold-free cluster enhancement (TFCE with 5,000 permutations, threshold at $p < 0.001$, and pFWE < 0.05 respectively).

Correction: FWE

Models & analysis

n/a	Involved in the study
☒	☐ Functional and/or effective connectivity
☒	☐ Graph analysis
☐	☒ Multivariate modeling or predictive analysis

Multivariate modeling and predictive analysis: The state of art machine-learning framework in neuroimaging (Kohoutova et al., 2020) was adopted to explore whether use of the different pipelines will affect prediction accuracy of sex and age by means of distributed brain structural variations (GMV maps). For the categorical prediction (sex) the 200 healthy participants from dataset 1 were divided into two sex- and age-matched independent samples which served as training and test datasets respectively. A support vector machine (SVM, C = 1) was employed to develop an MVPA-based sex classifier. The SVM was trained on the training data (n = 100) with a bootstrapping test to find stable features (5,000 permutations, pFDR < 0.05). Next these features were used to train the model by means of 5-fold cross-validation. The resulting patterns were subsequently tested in the independent test sample (n = 100) to determine within- and between-pipeline prediction accuracy for sex. To estimate the effect size of each classification Cohen's d for between-subject designs was employed (Lakens, 2013). For prediction of a continuous variable (age) a support vector regression (SVR, epsilon = 0.1, C = 1) model was trained on dataset 2. A bootstrapping test (5,000 permutations, pFDR < 0.05) was used to find stable features. These features and a 5-fold cross-validation were applied to train the model. Prediction performance was next quantified by evaluation of correlation strengths between predicted and true age for within- and between-pipelines.
